# A Simple Preparation of 2,3,4,6-Tetra-*O*-acyl-Gluco-, Galacto- and Mannopyranoses and Relevant Theoretical Study

**DOI:** 10.3390/molecules15010374

**Published:** 2010-01-18

**Authors:** Zerong Daniel Wang, Yirong Mo, Chiao-Lun Chiou, Minghong Liu

**Affiliations:** 1 Department of Chemistry, School of Science and Computer Engineering, University of Houston-Clear Lake, 2700 Bay Area Boulevard, Houston, TX 77058, USA; 2 Department of Chemistry, Western Michigan University, Kalamazuo, MI 49008, USA; 3 Department of Chemical & Biomolecular Engineering, University of Houston, Houston, TX 77204-4004, USA; 4 Chemistry Department, University of North Carolina-Charlotte, Charlotte, NC 28223, USA

**Keywords:** deacetylation, dealkylation, anomeric effect, deacylation, density functional theory

## Abstract

The deacylation of glucose, galactose and mannose pentaacetates, galactose and mannose penta(3-bromo)benzoates, as well as the dealkylation of 2,3,4,6-tetra-*O*-acetyl and 2,3,4,6-tetra-*O*-(3-bromo)benzoyl methyl α-*D*-glucopyranosides have been studied. In addition, a computational study on the deacylation of β-*D*-glucose pentaacetate has been carried out with density functional theory (B3LYP/6-31G*). The anomeric effect during deacetylation and dealkylation has been clearly demonstrated in both experimental and computational results.

## Introduction

A variety of biological activities of plants have been found to be associated with the presence of phenolic glycosides [1,2,3,4,5,6,7,8]. For example, *A. plantanifolium*, which contains the glycoside 2-(1-hydroxy-6-oxo-cyclohex-2-ene-1-carboxymethyl)-phenyl-4,6-*O*-[(*S*)-4,4’,5,5’,6,6’-hexahydroxydiphenoyl]-β-D-glucopyranose is used for the treatment of rheumatalgia, paralysis and cardianeuria [9], and is also used to promote the healing of wounds [10]. Also, the leaves of *Eriobotrya japonica *(Thunb.), containing a biphenyl glucoside, have been widely applied as a folk medicine (known as “Pipaye”) to treat stomachache and to promote antitussive, anti-inflammatory and diuretic effects in China [11]. In addition, the *Cucurbita* species (commonly known as pumpkin), being traditionally used for a deworming medicine (*C. moschata*) [12], and the treatment of urinary and prostate disease (*C. pepo*) [13], are found to contain many acylated phenolic glycosides [14]. Moreover, siebolsides A and B, which are isolated from the bark of *P. sieboldii*, have shown allergy preventive effects [15], and cypellocarpins A, B and C have *in vivo* antitumor-promoting effects [16]. Therefore, the preparation of phenolic glycosides has practical importance in order to further study the biological activity of this class of compounds. To our best knowledge, only a few preparations have been carried out for the direct glycosylation of phenol from monosaccharide peracetates or perbenzoates. For instance, the glucosides of phenols could be prepared directly through the fusion of glucose pentaacetate and phenols in the presence of ZnCl_2_ or *p*-toluenesulfonic acid [17]. Although this reaction has been shown to be impractical for the fusion of difficultly accessible phenols due to the need of an excess amount of phenols, moist phosphorus oxychloride has been applied to promote such fusion in benzene [18]. Other direct fusions of phenols include the preparation of phenyl 2,3,4,6-tetra-*O*-benzoyl-α-D-glucopyranose from D-glucose pentabenzoate and phenol in dichloromethane in the presence of BF_3_·Et_2_O [19], and the synthesis of 1-(4-nitrophenyl) 2,3,4,6-tetra-*O*-acetyl-α-D-mannopyranoside through the fusion of 1,2,3,4,6-penta-*O*-acetyl-β-D-mannopyranose and 4-nitrophenol in the presence of ZnCl_2_ [20]. However, when we carried out the fusion of glucose pentaacetate and 4-hydroxyacetophenone in the presence of ZnCl_2_or AlCl_3_(either in anhydrous Et_2_O or in the absence of solvent), we only isolated the product deacetylated at the anomeric position, *i.e.*, 2,3,4,6-tetra-*O*-acetyl-α-D-glucose. On the basis of this unexpected result, we extended our condition to different monosaccharide peresters as well as 2,3,4,6-tetra-*O*-acyl methyl glucosides. Reported here are the results for deacylation of glucose, mannose and galactose peresters, as well as the dealkylation of acylated methyl α-D-glucosides. Along with the experimental studies, a parallel computational study on the deacetylation of glucose pentaacetate has been performed using density functional theory (B3LYP/6-31G*).

## Results and Discussion

Initially, we planned to prepare phenyl glycosides in order to synthesize carbohydrate-containing styrenes for our glycopolymer project. When we attempted the reactions between β-D-glucose pentaacetate and 4-hydroxyacetophenone in the presence of ZnCl_2_, AlCl_3_ or *p*-toluenesulfonic acid by heating the reaction mixtures around 120–130 °C, no glycosylation product was observed. For example, in the presence of *p*-TsOH·H_2_O, the reaction mixture developed into a tar-like mass within one hour at a temperature of 120–130 °C. This substance had a very low solubility in EtOAc. Furthermore, the reaction mixture in the presence of anhydrous ZnCl_2_ melted at 107 °C and turned into a dark mixture, from which no glycosylation product was isolated by column chromatography except for a small amount of 4-acetylphenyl acetate. In order to promote the expected glycosylation, an even stronger Lewis acid (AlCl_3_) was applied. After heating the mixture of β-D-glucose pentaacetate and 4-hydroxyacetophenone in the presence of AlCl_3_ at 87 °C under vacuum for 4.5 hours, the mixture was fractionated by column chromatography to give two major products, *i.e.*, 4-acetylphenyl acetate (*R_f_* = 0.63, hexane/EtOAc = 1:1) and a sugar derivative of a higher polarity than β-D-glucose pentaacetate. This preliminary result indicates that at least one acyl group (e.g., acetyl) has been removed under the thermolytic condition with Lewis acid (such as AlCl_3_, ZnCl_2_, *etc*.) since the polarity of the resulting product is increased.

Therefore, a direct deacetylation of β-D-glucose pentaacetate in the presence of AlCl_3_ (2.25 equiv.) was carried out at 110 °C. No reaction was observed when the mixture was maintained at 94 °C or lower. Several hours later, the resulting black solid was pulverized and loaded directly to a short silica gel column and washed with hexane/EtOAc (5:1) to afford a pure compound, that was identified as 2,3,4,6-tetra-*O*-acetyl-α-D-glucopyranose (*R_f_* = 0.17, hexane/EtOAc = 3:1). In addition, this same reaction proceeded smoothly when the reaction mixture was heated under vacuum, and completed in a short period of time. Furthermore, when β-D-galactose pentaacetate and 2,3,4,6-tetra-*O*-acetyl methyl α-D-glucopyranoside were subjected to similar conditions, the corresponding 2,3,4,6-tetra-*O*-acetyl α-D-galacto- and α-D-glucopyranoses were obtained. When this procedure was extended to β-D-lactose octaacetate, the compound 2,3,4,6-tetra-*O*-acetyl-α-D-galactopyranose was identified, indicating the breakage of the glycosidic bond. 

For comparison, per(3-bromo)benzoates of α-D-lactose, α-D-glucose, α-D-galactose, α-D-mannose and β-D-mannose [21] were heated with AlCl_3_ at 110 °C. It was found that all of the anomeric acyl groups (*i.e.*, 3-bromobenzoyl) have been removed from the aforementioned per(3-bromo)benzoates. Moreover, all of the deacylation reactions have been repeated under the optimized condition, in which the respective carbohydrate peresters (either peracetate or per(3-bromo)benzoate) in 4–6 mL of anhydrous Et_2_O was heated with a small amount of AlCl_3_ at 110 °C (oven) in a Parr acid digestion bomb for 5 hours. Upon completion of the reaction, the mixture was loaded directly to a short column of silica gel and washed with hexane/EtOAc (8:1 for the reactions from (3-bromo)benzoates and 3:1 for reactions from acetates) to collect the product. However, when these reactions were carried out in a polar solvent, e.g., DMSO, DMF, two or more spots were identified by TLC and they were too close to be isolated in pure forms for structural characterization.

It is found in this study that all of the deacylations occur only at the anomeric position in anhydrous Et_2_O or in the absence of solvent, as shown in the deacylation of D-glucose (or D-galactose) peresters. In addition, alkyl protecting groups at the anomeric position can also be removed smoothly without affecting other acyl protecting groups on various hydroxyl groups of alkyl glycosides, as indicated by the breakage of the glycosidic bond in both D-lactose and methyl α-D-glucoside derivatives. In addition, the anomeric configuration for β-D-glucose and β-D-galactose derivatives have been inverted in the respective products, e.g., from β-D-glucose pentaacetate to 2,3,4,6-tetra-*O*-acetyl-α-D-glucopyranose. While α-D-glucose and α-D-galactose per(3-bromo)benzoates [21] also undergo a similar debenzoylation at the anomeric position, the original α-configuration is retained in the corresponding products. However, the debenzoylation of α-D-mannose per(3-bromo)benzoate affords 2,3,4,6-tetra-*O*-(3-bromo)benzoyl-β-D-mannopyranose, indicating the conversion of configuration at the anomeric position during the debenzoylation. The conversion of configuration at the anomeric site has been affirmed by the small NMR coupling constants between H-1 and H-2 in the resulting α-D-glucose (4.0– 4.1 Hz) and α-D-galactose (3.9 Hz) derivatives, which are consistent with the coupling constant of 3.6 Hz between H-1 and H-2 observed in either 2,3,4,6-tetra-*O*-acetyl methyl α-D-glucopyranoside or 2,3,4,6-tetra-*O*-(3-bromo)benzoyl methyl α-D-glucopyranoside prepared in this study, whereas the normal coupling constant between H-1 and H-2 in β-D-glucose derivatives was observed in the range of 7–8 Hz [22]. Likewise, an even smaller coupling constant between H-1 and H-2 for 2,3,4,6-tetra-*O*-(3-bromo)benzoyl D-mannose (1.2 Hz) strongly indicates the β-configuration at the anomeric site.

**Scheme 1:**
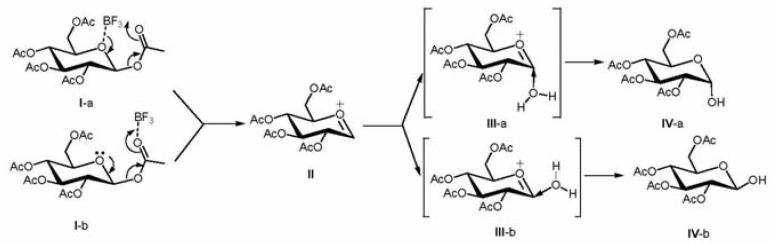
The possible reaction mechanism.

It is reasonably expected that an oxonium intermediate is generated in these reactions, onto which the attack of water affords the corresponding product during the workup stage (e.g., moisture in air or column). On the other hand, the facial preference during the attack of water molecule on the oxonium intermediate will determine the final geometry of the product. To rationalize the inversion of configuration at the anomeric position, DFT computations at the B3LYP/6-31G* level have been performed to derive the optimal geometries as well as energy variations for the deacetylation of β-*D*-glucose pentaacetate in the presence of BF_3_. Furthermore, possible reaction pathways between an oxonium intermediate and water were studied at the same level (**III**-a and **III**-b), and the bond energies between β-*D*-glucose pentaacetate and BF_3_ were also computed at MP2/6-31G* level with the geometries optimized at the B3LYP/6-31G* level [23], as shown in Scheme 1. The computational results in Table 1 clearly illustrate a weak interaction between BF_3_ and the oxygen atom on the ring of pyranoside or anomeric acetyl group. It is calculated that the molecular complex arising from the interaction between BF_3_ and the anomeric acetyl group (structure **I**-b) is slightly more stable than that formed by the pyranoside oxygen and BF_3_ (structure **I**-a). In addition, at the MP2/6-31G* level the bond energy between BF_3_ and the anomeric acetyl group is -5.4 kcal/mol, which is about 2.5 kcal/mol more exothermic than the interaction between BF_3_ and the pyranoside oxygen (Table 1). Assuming an equilibrium exists between these two interactions, then the former interaction is about 27.6 times favored than the interaction with pyranoside oxygen at 100 °C. Therefore, the anomeric acetyl group can be activated by a Lewis acid such as BF_3_. As a result, the glycosidic bond is weakened through the complexation. This trend is also clearly shown by the bond distance between oxygen and boron atoms, which varies from 1.76 Å to 2.44 Å, respectively, as shown in Table 1. 

**Table 1:** The optimal bonding distance (Å) and binding energies with the basis set superposition error (BSSE) corrections for β-*D*-glucose pentaacetate.

Structure	R(O···B)	Binding Energy (B3LYP/6-31G*)	Binding Energy (MP2/6-31G*)
**I**-a	2.44 Å	-0.2 Kcal/mol	-2.9 Kcal/mol
**I**-b	1.76 Å	-2.0 Kcal/mol	-5.4 Kcal/mol

It is proposed that upon deacetylation, the oxonium intermediate is formed, and the attack of water molecule on the oxonium intermediate either from the bottom (structure **III**-a) or top side (structure **III**-b), will lead to the formation of the corresponding 2,3,4,6-tetra-*O*-acetyl α- or β-glucopyranose, respectively. At the B3LYP/6-31G* level, computations show that the energy for the attack from the top is about 9.6 kcal/mol higher than the total energy of oxonium and water, whereas the bottom attack is 3.5 kcal/mol lower than the total energy of the oxonium intermediate and water combined, indicating that the formation of the α-anomer for the case of D-glucose is favored in kinetics. Interestingly, the gas phase free energy of 2,3,4,6-tetra-*O*-acetyl-β-D-glucopyranose is computed to be -1297.5029 a.u. (Table 2), whereas the corresponding free energy of 2,3,4,6-tetra-*O*-acetyl-α-D-glucopyranose is calculated to be -1297.5072 a.u., showing that the α-anomer is about 2.7 kcal/mol more stable than the β-anomer. Providing that the α-anomer and β-anomer can equilibrate under our reaction condition (110 ºC), then the yield of the α-anomer is expected to be about 35 times that of the β-anomer. Such a preference for α-D-glucopyranose is consistent with our experimental result that the deacetylation of both α-D-glucose and β-D-glucose pentaacetate either in anhydrous Et_2_O or in the absence of solvent gives 2,3,4,6-tetra-*O*-acetyl-β-glucopyranose. It should be pointed out that the order of free energies for α- and β-anomers in a polar solvent such as DMSO is reversed, since the β-anomer has a higher polarity (4.49 Debyes) and a larger solvation energy (-26.8 Kcal/mol). The higher solvation energy for the β-anomer over the α-anomer (-22.8 Kcal/mol) could reduce the preference to 1/5.91 in the formation of α-anomer if the reaction is carried out in such a polar solvent. This result also partially parallels our experimental result that the deacetylation of β-D-glucose pentaacetate in either DMSO or DMF gave two or more than two products as identified by TLC which are almost inseparable by column chromatography. 

**Table 2:** The computed parameters for 2,3,4,6-tetra-*O*-acetyl α- and β-D-glucopyranose.

Structure	E_(gas)_ (a.u.)	ΔG_(gas)_ (a.u.)	Solvation Energy in DMSO	ΔG_(DMSO) _(a.u.)	Dipole Moment (Debye)
**IV**-a	-1297.79767	-1297.5072	-22.8 Kcal/mol	-1297.5435	3.32
**IV**-b	-1297.97037	-1297.5029	-26.8 Kcal/mol	-1297.5456	4.49

## Experimental

### General

All chemicals and solvents were purchased from either Aldrich or Alfa Aesar and were used as received. ^1^H-NMR and ^13^C-NMR were recorded in CDCl_3_ on a Bruker Avance 600 MHz NMR spectrometer (at 600 Mz for ^1^H and 150 MHz for ^13^C, TMS as internal standard) at the Keck/IMD NMR center founded by the W. M. Keck Foundation and the University of Houston. Column chromatography was performed on silica gel using hexane/EtOAc mixtures as eluents. Thin-layer chromatography (TLC) was performed on silica gel (precoated silica gel plate F254, Merck) and detected both by UV and heating with 1.5% H_2_SO_4_ in EtOH. The high pressure acid digestion bomb (model No. 4746), equipped with a 23 mL PTFE cup (A255 AC), was purchased from the Parr Instrument Company. The high-speed shaker was a Wrist Action^TM^ Shaker (Model 75) purchased from Burrell Scientific (Pittsburg, PA, USA). The preparations of α-D-glucose and α-D-galactose peracetate, as well as α-D-glucose, α-D-galactose, α-D-lactose per(3-bromo)benzoates have been reported previously [21]. In contrast, β-D-glucose and β-D-galactose peracetates were prepared according to a known method [24].

All the computational work has been performed at the Department of Chemistry, Western Michigan University at Kalamazuo, using the Gaussian 03 package. Among these calculations, the optimal geometries of all the structures have been performed using DFT method at the B3LYP/6-31G* level, and bond energies between β-*D*-glucose pentaacetate and BF_3_ were computed at MP2/6-31G* level with the geometries optimized at the B3LYP/6-31G* level [23]. In addition, a part of computational work has also been repeated at University of Houston-Clear Lake on a SGI Altix 330 server using Gaussian 03 package [23]. All the bond lengths, bond angles and dihedral angles of computed geometries are provided in the supplemental information.

### The preparation of 2,3,4,6-tetra-O-acetyl methyl α-D-glucopyranoside

To a cold mixture of methyl α-*D*-glucopyranoside (1.0 g, Aldrich) and pyridine (3.0 mL) was added acetic anhydride (3.0 mL). The mixture was stirred overnight and then transferred to a separation funnel with EtOAc (50 mL). The solution was washed with 2 N HCl, saturated NaHCO_3_ and brine, dried over Na_2_SO_4_ and concentrated to afford 2.263 g of 2,3,4,6-tetra-*O*-acetyl methyl-α-D-glucopyranoside, which was further purified by crystallization in EtOAc/hexane, in almost quantitative yield. ^1^H-NMR (CDCl_3_): δ 5.47 (dd, 1H, *J*_2,3_ 10.0 Hz, *J*_3,4_ 9.6 Hz, H-3), 5.07 (dd, 1H, *J*_4,5_ 10.1 Hz, *J*_3,4_ 9.5 Hz, H-4), 4.96 (d, 1H, *J*_1,2_ 3.4 Hz, H-1), 4.90 (dd, 1H, *J*_2,3_ 10.2 Hz, *J*_1,2_ 3.6 Hz, H-2), 4.27 (dd, 1H, *J*_6,6’_ 12.3 Hz, *J*_5,6_ 4.6 Hz, H-6), 4.11 (dd, 1H, *J*_6,6’_ 12.3 Hz, *J*_5,6’_ 2.1 Hz, H-6’), 3.99 (ddd, 1H, *J*_4,5_ 9.0 Hz, *J*_5,6_ 4.6 Hz, *J*_5,6’_ 2.2 Hz, H-5), 3.42 (s, 3H, OMe), 2.10 (s, 3H, Ac), 2.08 (s, 3H, Ac), 2.03 (s, 3H, Ac), 2.01 (s, 3H, Ac); ^13^C-NMR (CDCl_3_): δ 170.50, 169.98, 169.91, 169.45, 96.64 (C-1), 70.64 (C-2), 69.97 (C-3), 68.40 (C-4), 67.00 (C-5), 61.79 (C-6), 55.31 (OMe).

### The preparation of 2,3,4,6-tetra-O-(3-bromo)benzoyl methyl α-D-glucopyranoside

To a 50 mL flask were added methyl α-D-glucopyranoside (0.529 g) and pyridine (4.0 mL). Then the flask was sealed by septum, vacuumed by a needle, and mounted to a high-speed shaker. After shaking for 10 minutes, the flask was wrapped with dry ice, then 3-bromobenzoyl chloride (2.0 mL, 5.6 equiv.) was added, and the mixture was left at -78ºC for 1 hour. Then the flask was shaken until all dry ice disappeared, and the light brown residue was transferred to a separation funnel, and worked up accordingly to afford 2.768 g of 2,3,4,6-tetra-*O*-(3-bromo)benzoyl methyl-α-D-glucopyranoside, in a yield of 91.2%. ^1^H-NMR (CDCl_3_): δ 8.15 (t, 1H, 1.6 Hz), 8.09 (t, 1H, 1.6 Hz), 8.04 (t, 1H, 1.7 Hz), 8.01 (t, 1H, 1.7 Hz), 7.97 (d, 1H, 7.8 Hz), 7.90 (d, 1H, 7.8 Hz), 7.85 (d, 1H, 7.8 Hz), 7.81 (d, 1H, 7.9 Hz), 7.68 (dt, 1H, J_o_ 7.1 Hz, *J*_m_ 0.8 Hz), 7.64 (dt, 1H, *J*_o_ 8.0 Hz, *J*_m_ 0.8 Hz), 7.63 (dt, 1H, *J*_o_ 8.8 Hz, *J*_m_ 0.8 Hz, 1H), 7.56 (dt, 1H, *J*_o_ 8.0 Hz, *J*_m_ 0.8 Hz), 7.31 (t, 1H, 7.8 Hz), 7.27 (t, 1H, 8.0 Hz), 7.24 (t, 1H, 7.9 Hz), 7.19 (t, 1H, 7.9 Hz), 6.14 (dd, 1H, J_2,3_ 9.8 Hz, *J*_3,4_ 9.8 Hz, H-3), 5.64 (dd, 1H, *J*_3,4_ 9.8 Hz, *J*_4,5_ 9.8 Hz, H-4), 5.29 (dd, 1H, *J*_1,2_ 3.6 Hz, *J*_2,3_ 10.1 Hz, H-2), 5.26 (d, 1H, *J*_1,2_ 3.6 Hz, H-1), 4.59 (dd, 1H, *J*_6,6’_ 12.1 Hz, *J*_5,6_ 3.2 Hz, H-6), 4.52 (dd, 1H, *J*_6,6’_ 12.1 Hz, *J*_5,6’_ 4.9 Hz, H-6’), 4.42-4.45 (m, 1H, H-5), 3.51 (s, 3H, OMe); ^13^C-NMR (CDCl_3_): δ 164.73, 164.44, 164.40, 163.91, 136.52, 136.43, 136.30, 136.14, 132.85, 132.69, 132.62, 131.37, 130.73, 130.48, 130.01, 128.37, 128.31, 128.19, 96.84 (C-1), 72.11 (C-2), 70.82 (C-3), 69.82 (C-4), 67.24 (C-5), 63.28 (C-6), 55.72 (OMe).

### The deacetylation of β-D-glucose pentaacetate

To a high pressure acid digestion bomb were added β-D-glucose pentaacetate (176.6 mg), Et_2_O (5 mL) and AlCl_3_ (60.3 mg, 1.0 equiv.). The reaction vessel was sealed and put in an oven at 110 ºC. After 4.5 hours, the ether solution was loaded directly to a silica gel column, and washed by hexane/EtOAc (3:1) to afford 99.9 mg of 2,3,4,6-tetra-*O*-acetyl-α-glucopyranose (63.4 % yield). *R_f_* = 0.17 (hexane/EtOAc = 3:1); ^1^H-NMR (CDCl_3_): δ 6.30 (d, 1H, J_1,2_ 4.0 Hz, H-1), 5.56 (dd, 1H, J_2,3_ 9.8 Hz, J_3,4_ 9.8 Hz, H-3), 5.14 (dd, 1H, J_3,4_ 9.8 Hz, J_4,5_ 9.8 Hz, H-4), 5.03 (dd, 1H, J_2,3_ 10.1 Hz, J_1,2_ 4.0 Hz, H-2), 4.30-4.34 (m, 2H, H-5, H-6), 4.13 (d, 1H, 10.8 Hz, H-6’), 2.105 (s, 3H, Ac), 2.101 (s, 3H, Ac), 2.05 (s, 3H, Ac), 2.04 (s, 3H, Ac); ^13^C-NMR (CDCl_3_): δ 170.39, 169.75 (2C), 169.35, 90.00 (C-1), 70.61 (C-2), 70.29 (C-5), 69.31 (C-3), 67.30 (C-4), 61.01 (C-6).

### The deacetylation of α-D-lactose octaacetate

To a 5 mL scintillation vial were added α-D-lactose octaacetate (0.144 g) and AlCl_3_ (0.273 g, 9.6 equiv.). The vial was swirled to mix the reactants well and left on a hot plate at 110 ºC. After one and a half hours, a weight of 14.04 mg was lost. The residue was transferred to a short silica gel column and washed by hexane/EtOAc (5:1 to 3:1) to afford 27.7 mg of 2,3,4,6-tetra-*O*-acetyl-α-D-galactopyranose, in a yield of 37.5%, *R_f_* = 0.14 (hexane/EtOAc = 3:1); ^1^H-NNR (CDCl_3_): δ 6.37 (d, 1H, J_1,2_ 3.9 Hz, H-1), 5.52 (d, 1H, 3.0 Hz, H-4), 5.42 (dd, 1H, J_2,3_ 10.7 Hz, J_3,4_ 3.3 Hz, H-3), 5.25 (dd, 1H, J_2,3_ 10.7 Hz, J_1,2_ 3.9 Hz, H-2), 4.52 (dd, 1H, J_5,6_ 6.6 Hz, J_5,6’_ 6.6 Hz, H-5), 4.17 (dd, 1H, J_6,6’_ 11.4 Hz, J_5,6_ 6.4 Hz, H-6), 4.11 (dd, 1H, J_6,6’_ 11.4 Hz, J_5,6’_ 6.7 Hz, H-6’), 2.15 (s, 3H, Ac), 2.11 (s, 3H, Ac), 2.06 (s, 3H, Ac), 2.01 (s, 3H, Ac); ^13^C-NMR (CDCl_3_): δ 170.32, 170.12, 169.92, 1369.78, 91.17 (C-1), 69.35 (C-5), 67.85 (C-2), 67.20 (C-4), 67.09 (C-3), 61.00 (C-6).

### The dealkylation of 2,3,4,6-tetra-O-(3-bromo)benzoyl methyl α-D-glucopyranoside

To a high pressure acid digestion bomb were added 2,3,4,6-tetra-*O*-(3-bromo)benzoyl methyl α-D-glucopyranoside (177.1 mg), AlCl_3_ (36.5 mg, 1.4 equiv.) and Et_2_O (4 mL). The reaction vessel was sealed and put in an oven at 110 ºC for 5 h, and the solution was loaded directly to a short silica gel column and washed with hexane/EtOAc (8:1) to afford 97.7 mg of 2,3,4,6-tetra-*O*-(3-bromobenzoyl)-α-D-glucopyranose, in a yield of 56.0%. *R_f_* = 0.39 (hexane/EtOAc = 3:1); ^1^H-NMR (CDCl_3_): δ 8.16 (d, 1H, 1.4 Hz), 8.10 (d, 1H, 1.4 Hz), 8.05 (d, 1H, 1.4 Hz), 8.01 (d, 1H, 1.4 Hz), 7.98 (dt, 1H, J_o_ 7.7 Hz, J_m_ 1.0 Hz), 7.91 (dt, 1H, J_o_ 7.9 Hz, J_m_ 1.0 Hz), 7.87 (dd, 1H, J_o_ 7.9 Hz, J_m_ 0.8 Hz), 7.81 (dd, 1H, J_o_ 7.9 Hz, J_m_ 0.9 Hz), 7.68 (dd, 1H, J_o_ 8.0 Hz, J_m_ 1.0 Hz), 7.66 (dd, 1H, J_o_ 8.0 Hz, J_m_ 1.0 Hz), 7.64 (dd, 1H, J_o_ 8.2 Hz, J_m_ 0.9 Hz), 7.58 (dd, 1H, J_o_ 8.0 Hz, J_m_ 1.0 Hz), 7.32 (t, 1H, 7.8 Hz), 7.28 (t, 1H, 8.3 Hz), 7.25 (t, 1H, 7.8 Hz), 7.19 (t, 1H, 7.9 Hz), 6.56 (d, 1H, J_1,2_ 4.0 Hz, H-1), 6.21 (dd, 1H, J_2,3_ 9.8 Hz, J_3,4_ 9.8 Hz, H-3), 5.75 (dd, 1H, J_3,4_ 9.8 Hz, J_4,5_ 10.1 Hz, H-4), 5.49 (dd, 1H, J_2,3_ 10.1 Hz, J_1,2_ 4.1 Hz, H-2), 4.77 (ddd, 1H, J_4,5_ 10.2 Hz, J_5,6_ 3.8 Hz, J_5,6’_ 3.2 Hz, H-5), 4.64 (dd, 1H, J_6,6’_ 12.5 Hz, J_5,6’_ 2.9 Hz, H-6’), 4.55 (dd, 1H, J_6,6’_ 12.5 Hz, J_5,6_ 4.3 Hz, H-6); ^13^C-NMR (CDCl_3_): δ 164.58, 164.25, 163.98, 163.74, 136.78, 136.69, 136.48, 136.23, 135.66, 132.89, 132.71, 132.64, 132.45, 131.14, 130.36, 130.10, 129.97, 129.82, 128.62, 128.43, 128.28, 128.20, 128.05, 122.61, 122.50, 89.97 (C-1), 71.72 (C-2), 70.52 (C-5), 70.24 (C-3), 68.53 (C-4), 62.32 (C-6).

### The deacylation of α-D-galactopyranose penta(3-bromo)benzoate

The mixture of α-D-galactopyranose penta(3-bromo)benzoate (305.9 mg), AlCl_3_ (41.0 mg, 1.1 equiv.) and Et_2_O (4 mL) in a high pressure acid digestion bomb was left in an oven for 5 h (110 ºC). After cooling down to room temperature, the solution was loaded directly to a short silica gel column and washed with hexane/EtOAc (5:1) to afford 177.4 mg of 2,3,4,6-tetra-*O*-(3-bromo)benzoyl-α-*D*-galactopyranose (69.6% yield), *R_f_* = 0.46 (hexane/EtOAc = 3:1); ^1^H-NMR (CDCl_3_): δ 8.17 (t, 1H, 1.4 Hz), 8.12 (t, 1H, 1.5 Hz), 8.11 (t, 1H, 1.6 Hz), 8.02 (t, 1H, 7.8 Hz), 7.92 (dd, 2H, J_o_ 7.8 Hz, J_m_ 1.0 Hz), 7.88 (t, 1H, 1.7 Hz), 7.76 (dt, 1H, J_o_ 8.0 Hz, J_m_ 0.9 Hz), 7.73 (d, 1H, 7.9 Hz), 7.67 (dd, 1H, J_o_ 6.9 Hz, J_m_ 0.9 Hz), 7.66 (dt, 1H, J_o_ 7.4 Hz, J_m_ 0.9 Hz), 7.59 (dt, 1H, J_o_ 7.4 Hz, J_m_ 1.0 Hz), 7.39 (t, 1H, 7.9 Hz), 7.30 (t, 1H, 7.9 Hz), 7.28 (t, 1H, 8.0 Hz), 7.17 (t, 1H, 7.9 Hz), 6.67 (d, 1H, J_1,2_ 3.9 Hz, H-1), 6.08 (d, 1H, J_3,4_ 3.3 Hz, H-4), 6.04 (dd, 1H, J_2,3_ 10.4 Hz, J_3,4_ 3.4 Hz, H-3), 5.78 (dd, 1H, J_2,3_ 10.34 Hz, J_1,2_ 3.9 Hz, H-2), 4.95 (dd, 1H, J_5,6_ 6.2 Hz, J_5,6’_ 6.1 Hz, H-5), 4.64 (dd, 1H, J_6,6’_ 11.6 Hz, J_5,6_ 6.7 Hz, H-6), 4.50 (dd, 1H, J_6,6’_ 11.6 Hz, J_5,6’_ 6.1 Hz, H-6’); ^13^C-NMR (CDCl_3_): δ 164.49, 164.21, 164.19, 163.90, 136.89, 136.75, 136.44, 136.30, 132.79, 132.74, 132.66, 132.62, 130.96, 130.45, 130.41, 130.33, 130.27, 130.09, 129.97, 128.35, 128.24, 128.16, 122.91, 122.60, 122.49, 122.45, 90.98 (C-1), 69.69(C-5), 68.98 (C-2), 68.58 (C-4), 68.14 (C-3), 61.92 (C-6).

### The deacylation of α-D-mannose penta(3-bromo)benzoate

To a high pressure acid digestion bomb were added α-D-mannose penta(3-bromo)benzoate (142.1 mg), AlCl_3_ (61.0 mg, 3.5 equiv.) and Et_2_O (5 mL). After reaction at 110ºC for 4 hours, the solution was loaded directly to a short silica gel column and washed with hexane/EtOAc (5:1) to give 103.0 mg of 2,3,4,6-tetra-*O*-(3-bromo)benzoyl-α-D-mannopyranose (87.0% yield), *R_f_* = 0.48 (hexane/EtOAc = 3:1); ^1^H-NMR (CDCl_3_): δ 8.17 (t, 1H, 1.6 Hz), 8.15 (t, 1H, 1.6 Hz), 8.08 (t, 1H, 1.6 Hz), 7.98 (dd, 1H, J_o_ 7.7 Hz, J_m_ 1.0 Hz), 7.94 (dd, 1H, J_o_ 7.8 Hz, J_m_ 0.9 Hz), 7.92 (t, 1H, 1.6 Hz), 7.90 (dd, 1H, J_o_ 7.8 Hz, J_m_ 0.9 Hz), 7.75 (ddd, 1H, J_o_ 8.0 Hz, J_m_ 1.8 Hz, J_p_ 0.9 Hz), 7.73 (dt, 1H, J_o_ 7.0 Hz, J_m_ 0.9 Hz), 7.70 (dt, 1H, J_o_ 8.0 Hz, J_m_ 0.9 Hz), 7.66 (dt, 1H, J_o_ 8.0 Hz, J_m_ 0.8 Hz), 7.60 (dt, 1H, J_o_ 8.0 Hz, J_m_ 0.9 Hz), 7.303 (t, 1H, 7.9 Hz), 7.298 (t, 1H, 7.7 Hz), 7.27 (t, 1H, 7.8 Hz), 7.18 (t, 1H, 7.9 Hz), 6.27 (d, 1H, J_1,2_ 1.2 Hz, H-1), 6.13 (dd, 1H, J_3,4_ 10.2 Hz, J_2,3_ 3.3 Hz, H-3), 6.03 (dd, 1H, J_3,4_ 10.1 Hz, J_4,5_ 10.1 Hz, H-4), 5.84 (dd, 1H, J_2,3_ 3.2 Hz, J_1,2_ 1.7 Hz, H-2), 4.74 (ddd, 1H, J_4,5_ 9.8 Hz, J_5,6_ 3.4 Hz, J_5,6’_ 3.2 Hz, H-5), 4.70 (dd, 1H, J_6,6’_ 12.4 Hz, J_5,6’_ 2.8 Hz, H-6’), 4.56 (dd, 1H, J_6,6’_ 12.4 Hz, J_5,6_ 4.1 Hz, H-6); ^13^C-NMR (CDCl_3_): δ 164.59, 163.98, 163.91, 163.87, 136.92, 136.74, 136.53, 136.24, 132.95, 132.72, 131.33, 130.37, 130.09, 130.03, 128.37, 128.15, 122.91, 122.67, 122.61, 122.53, 88.57 (C-1), 72.64 (C-2), 71.15 (C-5), 69.06 (C-3), 66.57 (C-4), 62.45 (C-6), 20.54, 20.49, 20.45 (2C).

## Conclusions

Both experimental and theoretical studies show that the deacetylation of α- or β-*D*-glucose pentaacetate by the action of Lewis acid will give 2,3,4,6-tetra-*O*-acetyl-α-D-glucopyranose. In addition, experimental results also indicate that the deacylation of D-galactose peresters will give results similar to that of D-glucose perester, whereas the corresponding debenzoylation of α-D-mannose per(3-bromo)benzoate will afford 2,3,4,6-tetra-*O*-(3-bromo)benzoyl β-D-mannopyranose. 

## Supplementary Materials

Supplementary information can be seen at http://www.mdpi.com/1420-3049/15/1/374/s1/.
