# Supplementary material for: A Simple Preparation of 2,3,4,6-Tetra-O-acyl-Gluco-, Galacto- and Mannopyranoses and Relevant Theoretical Study
Source: Molecules. 2010 Jan 18;15(1):374–84. doi: 10.3390/molecules15010374 (PMC6257110; doi:10.3390/molecules15010374)
Supplement: Supplementary file 1 [file molecules-15-00374-s001.pdf]

Supplementary Information

A Simple Preparation of 2,3,4,6-Tetra-*O*-acyl-Gluco-, Galacto- and Mannopyranoses and Relevant Theoretical Study

Zerong Daniel Wang[1,]*, Yirong Mo[2,]*, Chiao-Lun Chiou[1,3], and Minghong Liu[2,4]

[1] Department of Chemistry, School of Science and Computer Engineering, University of Houston-Clear Lake, 2700 Bay Area Boulevard, Houston, TX 77058, USA

[2] Department of Chemistry, Western Michigan University, Kalamazuo, MI 49008, USA

[3] Department of Chemical & Biomolecular Engineering, University of Houston, Houston, TX 77204-4004, USA

[4] ouHoustonHHChemistry Department, University of North Carolina-Charlotte, Charlotte, NC 28223, USA

Structure I-a

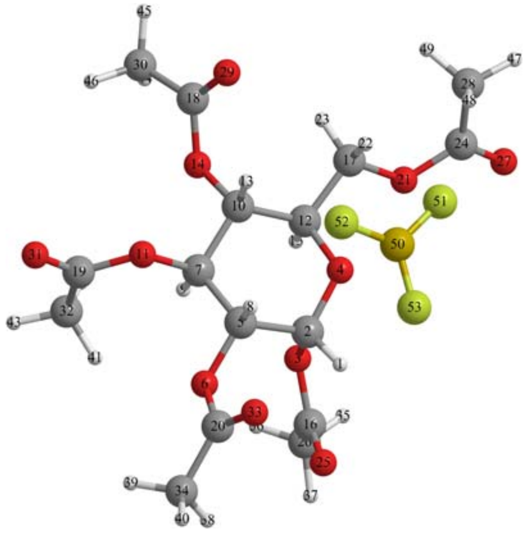

Bond	Bond Length (Å)	Bond Angle	Value (º)	Dihedral Angle	Value (º)
O(4)-B(50)	2.44	F(53)-B(50)-F(52)	120.15	H(47)-C(28)-C(24)-O(21)	-175.21
F(51)-B(50)	1.33	F(53)-B(50)-F(51)	119.97	H(47)-C(28)-C(24)-O(27)	5.56
F(52)-B(50)	1.33	F(52)-B(50)-F(51)	119.25	H(48)-C(28)-C(24)-O(21)	65.54
F(53)-B(50)	1.32	H(40)-C(34)-H(39)	110.48	H(48)-C(28)-C(24)-O(27)	-113.69
H(38)-C(34)	1.10	H(40)-C(34)-H(38)	109.16	H(49)-C(28)-C(24)-O(21)	-54.59
H(39)-C(34)	1.09	H(40)-C(34)-C(20)	109.33	H(49)-C(28)-C(24)-O(27)	126.18
H(40)-C(34)	1.09	H(39)-C(34)-H(38)	108.20	O(27)-C(24)-O(21)-C(17)	-176.29
H(41)-C(32)	1.09	H(39)-C(34)-C(20)	111.29	C(28)-C(24)-O(21)-C(17)	4.44
H(42)-C(32)	1.10	H(38)-C(34)-C(20)	108.32	H(38)-C(34)-C(20)-O(6)	75.75
H(43)-C(32)	1.09	H(43)-C(32)-H(42)	108.91	H(38)-C(34)-C(20)-O(33)	-102.78

H(44)-C(30)	1.09	H(43)-C(32)-H(41)	109.68	H(39)-C(34)-C(20)-O(6)	-43.08
H(45)-C(30)	1.09	H(43)-C(32)-C(19)	107.50	H(39)-C(34)-C(20)-O(33)	138.40
H(46)-C(30)	1.10	H(42)-C(32)-H(41)	108.15	H(40)-C(34)-C(20)-O(6)	-165.40
H(47)-C(28)	1.09	H(42)-C(32)-C(19)	111.23	H(40)-C(34)-C(20)-O(33)	16.07
H(48)-C(28)	1.10	H(41)-C(32)-C(19)	111.35	H(41)-C(32)-C(19)-O(11)	56.85
H(49)-C(28)	1.10	H(46)-C(30)-H(45)	109.24	H(41)-C(32)-C(19)-O(31)	-123.40
H(35)-C(26)	1.10	H(46)-C(30)-H(44)	107.62	H(42)-C(32)-C(19)-O(11)	-63.86
H(36)-C(26)	1.09	H(46)-C(30)-C(18)	109.24	H(42)-C(32)-C(19)-O(31)	115.89
H(37)-C(26)	1.09	H(45)-C(30)-H(44)	110.47	H(43)-C(32)-C(19)-O(11)	177.00
O(27)-C(24)	1.21	H(45)-C(30)-C(18)	109.45	H(43)-C(32)-C(19)-O(31)	-3.26
C(28)-C(24)	1.52	H(44)-C(30)-C(18)	110.79	H(44)-C(30)-C(18)-O(14)	-46.00
C(24)-O(21)	1.37	H(49)-C(28)-H(48)	107.76	H(44)-C(30)-C(18)-O(29)	134.10
O(33)-C(20)	1.21	H(49)-C(28)-H(47)	109.44	H(45)-C(30)-C(18)-O(14)	-168.05
C(34)-C(20)	1.50	H(49)-C(28)-C(24)	112.12	H(45)-C(30)-C(18)-O(29)	12.05
O(31)-C(19)	1.20	H(48)-C(28)-H(47)	109.18	H(46)-C(30)-C(18)-O(14)	72.38
C(32)-C(19)	1.52	H(48)-C(28)-C(24)	110.36	H(46)-C(30)-C(18)-O(29)	-107.51
O(29)-C(18)	1.21	H(47)-C(28)-C(24)	107.95	C(24)-O(21)-C(17)-C(12)	-163.63
C(30)-C(18)	1.51	H(37)-C(26)-H(36)	110.13	C(24)-O(21)-C(17)-H(22)	-42.97
O(21)-C(17)	1.43	H(37)-C(26)-H(35)	109.78	C(24)-O(21)-C(17)-H(23)	77.82
H(22)-C(17)	1.09	H(37)-C(26)-C(16)	109.42	H(35)-C(26)-C(16)-O(3)	63.92
H(23)-C(17)	1.09	H(36)-C(26)-H(35)	107.44	H(35)-C(26)-C(16)-O(25)	-115.89
O(25)-C(16)	1.21	H(36)-C(26)-C(16)	110.39	H(36)-C(26)-C(16)-O(3)	-54.26
C(26)-C(16)	1.51	H(35)-C(26)-C(16)	109.66	H(36)-C(26)-C(16)-O(25)	125.92
C(18)-O(14)	1.37	C(28)-C(24)-O(27)	123.92	H(37)-C(26)-C(16)-O(3)	-175.62
H(15)-C(12)	1.10	C(28)-C(24)-O(21)	118.00	H(37)-C(26)-C(16)-O(25)	4.57
C(17)-C(12)	1.52	O(27)-C(24)-O(21)	118.08	O(29)-C(18)-O(14)-C(10)	9.44
C(19)-O(11)	1.37	C(24)-O(21)-C(17)	122.01	C(30)-C(18)-O(14)-C(10)	-170.46
H(13)-C(10)	1.09	C(34)-C(20)-O(33)	126.43	O(21)-C(17)-C(12)-O(4)	67.82
O(14)-C(10)	1.44	C(34)-C(20)-O(6)	110.59	O(21)-C(17)-C(12)-C(10)	-171.60
C(12)-C(10)	1.54	O(33)-C(20)-O(6)	122.96	O(21)-C(17)-C(12)-H(15)	-49.10
H(9)-C(7)	1.09	C(32)-C(19)-O(31)	123.54	H(22)-C(17)-C(12)-O(4)	-54.08
O(11)-C(7)	1.43	C(32)-C(19)-O(11)	118.71	H(22)-C(17)-C(12)-C(10)	66.49
C(10)-C(7)	1.53	O(31)-C(19)-O(11)	117.74	H(22)-C(17)-C(12)-H(15)	-171.01
C(20)-O(6)	1.38	C(30)-C(18)-O(29)	126.09	H(23)-C(17)-C(12)-O(4)	-173.25
O(6)-C(5)	1.43	C(30)-C(18)-O(14)	110.53	H(23)-C(17)-C(12)-C(10)	-52.67
H(8)-C(5)	1.09	O(29)-C(18)-O(14)	123.38	H(23)-C(17)-C(12)-H(15)	69.83
C(7)-C(5)	1.53	C(26)-C(16)-O(25)	126.12	O(31)-C(19)-O(11)-C(7)	-179.13
C(12)-O(4)	1.45	C(26)-C(16)-O(3)	110.33	C(32)-C(19)-O(11)-C(7)	0.62
C(16)-O(3)	1.37	O(25)-C(16)-O(3)	123.55	C(18)-O(14)-C(10)-C(7)	136.80
O(3)-C(2)	1.43	H(23)-C(17)-H(22)	108.45	C(18)-O(14)-C(10)-C(12)	-103.38
C(5)-C(2)	1.54	H(23)-C(17)-O(21)	110.02	C(18)-O(14)-C(10)-H(13)	18.18
O(4)-C(2)	1.41	H(23)-C(17)-C(12)	109.44	O(4)-C(12)-C(10)-C(7)	-55.15
C(2)-H(1)	1.09	H(22)-C(17)-O(21)	112.14	O(4)-C(12)-C(10)-O(14)	-171.97
		H(22)-C(17)-C(12)	110.19	O(4)-C(12)-C(10)-H(13)	66.96
		O(21)-C(17)-C(12)	106.57	C(17)-C(12)-C(10)-C(7)	-174.78
		C(18)-O(14)-C(10)	117.42	C(17)-C(12)-C(10)-O(14)	68.40
		H(15)-C(12)-C(17)	108.51	C(17)-C(12)-C(10)-H(13)	-52.67
		H(15)-C(12)-C(10)	109.80	H(15)-C(12)-C(10)-C(7)	63.43
		H(15)-C(12)-O(4)	108.40	H(15)-C(12)-C(10)-O(14)	-53.39

		C(17)-C(12)-C(10)	113.73	H(15)-C(12)-C(10)-H(13)	-174.45
		C(17)-C(12)-O(4)	107.30	C(19)-O(11)-C(7)-C(5)	-110.05
		C(10)-C(12)-O(4)	108.96	C(19)-O(11)-C(7)-C(10)	129.91
		C(19)-O(11)-C(7)	123.69	C(19)-O(11)-C(7)-H(9)	11.07
		H(13)-C(10)-O(14)	109.43	C(12)-C(10)-C(7)-C(5)	52.35
		H(13)-C(10)-C(12)	110.26	C(12)-C(10)-C(7)-O(11)	171.69
		H(13)-C(10)-C(7)	110.14	C(12)-C(10)-C(7)-H(9)	-67.82
		O(14)-C(10)-C(12)	110.46	O(14)-C(10)-C(7)-C(5)	172.02
		O(14)-C(10)-C(7)	105.78	O(14)-C(10)-C(7)-O(11)	-68.64
		C(12)-C(10)-C(7)	110.69	O(14)-C(10)-C(7)-H(9)	51.85
		H(9)-C(7)-O(11)	111.28	H(13)-C(10)-C(7)-C(5)	-69.84
		H(9)-C(7)-C(10)	108.70	H(13)-C(10)-C(7)-O(11)	49.50
		H(9)-C(7)-C(5)	109.47	H(13)-C(10)-C(7)-H(9)	170.00
		O(11)-C(7)-C(10)	107.38	O(33)-C(20)-O(6)-C(5)	3.72
		O(11)-C(7)-C(5)	109.45	C(34)-C(20)-O(6)-C(5)	-174.87
		C(10)-C(7)-C(5)	110.54	C(20)-O(6)-C(5)-C(2)	86.88
		C(20)-O(6)-C(5)	115.98	C(20)-O(6)-C(5)-C(7)	-151.86
		H(8)-C(5)-C(7)	110.15	C(20)-O(6)-C(5)-H(8)	-31.92
		H(8)-C(5)-O(6)	109.57	C(10)-C(7)-C(5)-C(2)	-51.47
		H(8)-C(5)-C(2)	108.15	C(10)-C(7)-C(5)-O(6)	-172.16
		C(7)-C(5)-O(6)	107.92	C(10)-C(7)-C(5)-H(8)	68.26
		C(7)-C(5)-C(2)	110.99	O(11)-C(7)-C(5)-C(2)	-169.56
		O(6)-C(5)-C(2)	110.07	O(11)-C(7)-C(5)-O(6)	69.76
		C(12)-O(4)-C(2)	114.68	O(11)-C(7)-C(5)-H(8)	-49.82
		C(16)-O(3)-C(2)	117.85	H(9)-C(7)-C(5)-C(2)	68.23
		H(1)-C(2)-C(5)	111.17	H(9)-C(7)-C(5)-O(6)	-52.45
		H(1)-C(2)-O(4)	107.19	H(9)-C(7)-C(5)-H(8)	-172.03
		H(1)-C(2)-O(3)	109.66	C(10)-C(12)-O(4)-C(2)	61.22
		C(5)-C(2)-O(4)	110.21	C(17)-C(12)-O(4)-C(2)	-175.23
		C(5)-C(2)-O(3)	110.15	H(15)-C(12)-O(4)-C(2)	-58.24
		O(4)-C(2)-O(3)	108.37	O(25)-C(16)-O(3)-C(2)	6.25
				C(26)-C(16)-O(3)-C(2)	-173.57
				C(16)-O(3)-C(2)-O(4)	137.03
				C(16)-O(3)-C(2)-C(5)	-102.34
				C(16)-O(3)-C(2)-H(1)	20.32
				O(6)-C(5)-C(2)-O(3)	54.44
				O(6)-C(5)-C(2)-O(4)	173.97
				O(6)-C(5)-C(2)-H(1)	-67.33
				C(7)-C(5)-C(2)-O(3)	-64.97
				C(7)-C(5)-C(2)-O(4)	54.56
				C(7)-C(5)-C(2)-H(1)	173.26
				H(8)-C(5)-C(2)-O(3)	174.10
				H(8)-C(5)-C(2)-O(4)	-66.37
				H(8)-C(5)-C(2)-H(1)	52.33
				C(12)-O(4)-C(2)-O(3)	59.73
				C(12)-O(4)-C(2)-C(5)	-60.88
				C(12)-O(4)-C(2)-H(1)	178.02

Structure I-b

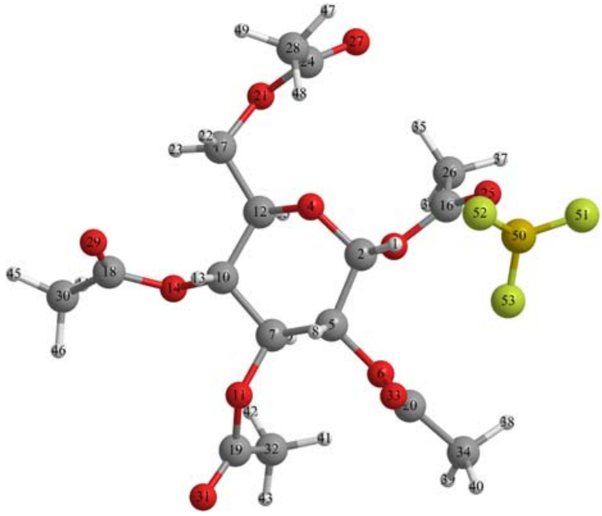

Bond	Bond Length (Å)	Bond Angle	Value (º)	Dihedral Angle	Value (º)
F(51)-B(50)	1.34	F(51)-B(50)-F(52)	116.96	H(49)-C(28)-C(24)-O(21)	-45.31
F(52)-B(50)	1.36	F(51)-B(50)-F(53)	116.31	H(49)-C(28)-C(24)-O(27)	131.25
F(53)-B(50)	1.37	F(52)-B(50)-F(53)	114.95	H(48)-C(28)-C(24)-O(21)	75.15
H(38)-C(34)	1.09	H(38)-C(34)-H(39)	108.35	H(48)-C(28)-C(24)-O(27)	-108.29
H(39)-C(34)	1.09	H(38)-C(34)-H(40)	109.36	H(47)-C(28)-C(24)-O(21)	-165.96
H(40)-C(34)	1.09	H(38)-C(34)-C(20)	107.91	H(47)-C(28)-C(24)-O(27)	10.60
H(41)-C(32)	1.09	H(39)-C(34)-H(40)	110.53	C(28)-C(24)-O(21)-C(17)	-33.60
H(42)-C(32)	1.10	H(39)-C(34)-C(20)	111.33	O(27)-C(24)-O(21)-C(17)	149.63
H(43)-C(32)	1.09	H(40)-C(34)-C(20)	109.31	H(40)-C(34)-C(20)-O(6)	-164.48
H(44)-C(30)	1.09	H(41)-C(32)-H(42)	108.17	H(40)-C(34)-C(20)-O(33)	16.52
H(45)-C(30)	1.09	H(41)-C(32)-H(43)	109.62	H(39)-C(34)-C(20)-O(6)	-42.09
H(46)-C(30)	1.10	H(41)-C(32)-C(19)	111.46	H(39)-C(34)-C(20)-O(33)	138.91
H(47)-C(28)	1.09	H(42)-C(32)-H(43)	108.87	H(38)-C(34)-C(20)-O(6)	76.69
H(48)-C(28)	1.09	H(42)-C(32)-C(19)	111.20	H(38)-C(34)-C(20)-O(33)	-102.32
H(49)-C(28)	1.10	H(43)-C(32)-C(19)	107.48	H(43)-C(32)-C(19)-O(11)	175.69
H(35)-C(26)	1.10	H(44)-C(30)-H(45)	110.45	H(43)-C(32)-C(19)-O(31)	-4.11
H(36)-C(26)	1.09	H(44)-C(30)-H(46)	107.60	H(42)-C(32)-C(19)-O(11)	-65.25
H(37)-C(26)	1.09	H(44)-C(30)-C(18)	110.79	H(42)-C(32)-C(19)-O(31)	114.95
O(27)-C(24)	1.21	H(45)-C(30)-H(46)	109.29	H(41)-C(32)-C(19)-O(11)	55.55
C(28)-C(24)	1.51	H(45)-C(30)-C(18)	109.44	H(41)-C(32)-C(19)-O(31)	-124.25
C(24)-O(21)	1.37	H(46)-C(30)-C(18)	109.24	H(46)-C(30)-C(18)-O(14)	72.23
O(33)-C(20)	1.20	H(47)-C(28)-H(48)	108.79	H(46)-C(30)-C(18)-O(29)	-107.50
C(34)-C(20)	1.50	H(47)-C(28)-H(49)	109.75	H(45)-C(30)-C(18)-O(14)	-168.16
O(31)-C(19)	1.20	H(47)-C(28)-C(24)	108.05	H(45)-C(30)-C(18)-O(29)	12.11
C(32)-C(19)	1.52	H(48)-C(28)-H(49)	108.32	H(44)-C(30)-C(18)-O(14)	-46.13
O(29)-C(18)	1.21	H(48)-C(28)-C(24)	110.46	H(44)-C(30)-C(18)-O(29)	134.14
C(30)-C(18)	1.51	H(49)-C(28)-C(24)	111.43	C(24)-O(21)-C(17)-C(12)	-85.96
O(21)-C(17)	1.43	H(35)-C(26)-H(36)	108.46	C(24)-O(21)-C(17)-H(23)	155.58
H(22)-C(17)	1.09	H(35)-C(26)-H(37)	108.89	C(24)-O(21)-C(17)-H(22)	36.89
H(23)-C(17)	1.09	H(35)-C(26)-C(16)	106.85	H(37)-C(26)-C(16)-O(3)	-154.96
O(25)-C(16)	1.23	H(36)-C(26)-H(37)	111.28	H(37)-C(26)-C(16)-O(25)	27.09
C(26)-C(16)	1.50	H(36)-C(26)-C(16)	111.08	H(36)-C(26)-C(16)-O(3)	-31.19

C(18)-O(14)	1.37	H(37)-C(26)-C(16)	110.16	H(36)-C(26)-C(16)-O(25)	150.85
H(15)-C(12)	1.10	O(27)-C(24)-C(28)	123.87	H(35)-C(26)-C(16)-O(3)	86.92
C(17)-C(12)	1.52	O(27)-C(24)-O(21)	117.89	H(35)-C(26)-C(16)-O(25)	-91.04
C(19)-O(11)	1.37	C(28)-C(24)-O(21)	118.15	C(30)-C(18)-O(14)-C(10)	-170.88
H(13)-C(10)	1.09	C(24)-O(21)-C(17)	122.26	O(29)-C(18)-O(14)-C(10)	8.86
O(14)-C(10)	1.44	O(33)-C(20)-C(34)	126.63	H(23)-C(17)-C(12)-O(4)	-171.09
C(12)-C(10)	1.53	O(33)-C(20)-O(6)	122.97	H(23)-C(17)-C(12)-C(10)	-49.87
H(9)-C(7)	1.09	C(34)-C(20)-O(6)	110.40	H(23)-C(17)-C(12)-H(15)	72.64
O(11)-C(7)	1.43	O(31)-C(19)-C(32)	123.65	H(22)-C(17)-C(12)-O(4)	-50.02
C(10)-C(7)	1.53	O(31)-C(19)-O(11)	117.68	H(22)-C(17)-C(12)-C(10)	71.19
C(20)-O(6)	1.38	C(32)-C(19)-O(11)	118.67	H(22)-C(17)-C(12)-H(15)	-166.29
O(6)-C(5)	1.43	O(29)-C(18)-C(30)	126.20	O(21)-C(17)-C(12)-O(4)	73.74
H(8)-C(5)	1.09	O(29)-C(18)-O(14)	123.12	O(21)-C(17)-C(12)-C(10)	-165.04
C(7)-C(5)	1.53	C(30)-C(18)-O(14)	110.68	O(21)-C(17)-C(12)-H(15)	-42.52
C(12)-O(4)	1.44	O(25)-C(16)-C(26)	121.20	C(32)-C(19)-O(11)-C(7)	3.85
C(16)-O(3)	1.33	O(25)-C(16)-O(3)	125.51	O(31)-C(19)-O(11)-C(7)	-176.34
O(3)-C(2)	1.46	C(26)-C(16)-O(3)	113.25	C(18)-O(14)-C(10)-C(7)	143.93
C(5)-C(2)	1.53	O(21)-C(17)-H(22)	112.81	C(18)-O(14)-C(10)-C(12)	-98.55
O(4)-C(2)	1.40	O(21)-C(17)-H(23)	105.94	C(18)-O(14)-C(10)-H(13)	24.39
C(2)-H(1)	1.09	O(21)-C(17)-C(12)	107.10	O(4)-C(12)-C(10)-C(7)	-60.13
		H(22)-C(17)-H(23)	108.60	O(4)-C(12)-C(10)-O(14)	-176.11
		H(22)-C(17)-C(12)	111.34	O(4)-C(12)-C(10)-H(13)	61.78
		H(23)-C(17)-C(12)	110.92	C(17)-C(12)-C(10)-C(7)	179.96
		C(18)-O(14)-C(10)	116.98	C(17)-C(12)-C(10)-O(14)	63.99
		H(15)-C(12)-C(17)	106.59	C(17)-C(12)-C(10)-H(13)	-58.13
		H(15)-C(12)-C(10)	108.64	H(15)-C(12)-C(10)-C(7)	58.49
		H(15)-C(12)-O(4)	109.34	H(15)-C(12)-C(10)-O(14)	-57.49
		C(17)-C(12)-C(10)	118.14	H(15)-C(12)-C(10)-H(13)	-179.60
		C(17)-C(12)-O(4)	105.68	C(19)-O(11)-C(7)-C(5)	-115.04
		C(10)-C(12)-O(4)	108.21	C(19)-O(11)-C(7)-C(10)	124.43
		C(19)-O(11)-C(7)	123.69	C(19)-O(11)-C(7)-H(9)	5.50
		H(13)-C(10)-O(14)	109.49	C(12)-C(10)-C(7)-C(5)	53.88
		H(13)-C(10)-C(12)	110.93	C(12)-C(10)-C(7)-O(11)	172.82
		H(13)-C(10)-C(7)	110.85	C(12)-C(10)-C(7)-H(9)	-66.43
		O(14)-C(10)-C(12)	111.22	O(14)-C(10)-C(7)-C(5)	173.28
		O(14)-C(10)-C(7)	105.86	O(14)-C(10)-C(7)-O(11)	-67.78
		C(12)-C(10)-C(7)	108.38	O(14)-C(10)-C(7)-H(9)	52.97
		H(9)-C(7)-O(11)	111.33	H(13)-C(10)-C(7)-C(5)	-68.08
		H(9)-C(7)-C(10)	108.43	H(13)-C(10)-C(7)-O(11)	50.86
		H(9)-C(7)-C(5)	109.45	H(13)-C(10)-C(7)-H(9)	171.62
		O(11)-C(7)-C(10)	108.01	C(34)-C(20)-O(6)-C(5)	-176.23
		O(11)-C(7)-C(5)	108.56	O(33)-C(20)-O(6)-C(5)	2.81
		C(10)-C(7)-C(5)	111.07	C(20)-O(6)-C(5)-C(2)	100.71
		C(20)-O(6)-C(5)	116.40	C(20)-O(6)-C(5)-C(7)	-135.16
		O(6)-C(5)-H(8)	109.11	C(20)-O(6)-C(5)-H(8)	-16.42
		O(6)-C(5)-C(7)	108.45	C(10)-C(7)-C(5)-C(2)	-47.94
		O(6)-C(5)-C(2)	110.89	C(10)-C(7)-C(5)-H(8)	70.22
		H(8)-C(5)-C(7)	109.12	C(10)-C(7)-C(5)-O(6)	-171.04
		H(8)-C(5)-C(2)	106.61	O(11)-C(7)-C(5)-C(2)	-166.55

		C(7)-C(5)-C(2)	112.61	O(11)-C(7)-C(5)-H(8)	-48.39
		C(12)-O(4)-C(2)	115.22	O(11)-C(7)-C(5)-O(6)	70.35
		C(16)-O(3)-C(2)	121.22	H(9)-C(7)-C(5)-C(2)	71.75
		O(3)-C(2)-C(5)	109.35	H(9)-C(7)-C(5)-H(8)	-170.08
		O(3)-C(2)-O(4)	107.43	H(9)-C(7)-C(5)-O(6)	-51.34
		O(3)-C(2)-H(1)	110.34	C(10)-C(12)-O(4)-C(2)	64.84
		C(5)-C(2)-O(4)	110.89	C(17)-C(12)-O(4)-C(2)	-167.70
		C(5)-C(2)-H(1)	111.88	H(15)-C(12)-O(4)-C(2)	-53.33
		O(4)-C(2)-H(1)	106.83	C(26)-C(16)-O(3)-C(2)	-146.24
				O(25)-C(16)-O(3)-C(2)	31.61
				C(16)-O(3)-C(2)-H(1)	-26.31
				C(16)-O(3)-C(2)-O(4)	89.79
				C(16)-O(3)-C(2)-C(5)	-149.78
				C(7)-C(5)-C(2)-H(1)	167.09
				C(7)-C(5)-C(2)-O(4)	47.93
				C(7)-C(5)-C(2)-O(3)	-70.36
				H(8)-C(5)-C(2)-H(1)	47.46
				H(8)-C(5)-C(2)-O(4)	-71.70
				H(8)-C(5)-C(2)-O(3)	170.01
				O(6)-C(5)-C(2)-H(1)	-71.19
				O(6)-C(5)-C(2)-O(4)	169.65
				O(6)-C(5)-C(2)-O(3)	51.37
				C(12)-O(4)-C(2)-H(1)	-179.87
				C(12)-O(4)-C(2)-C(5)	-57.72
				C(12)-O(4)-C(2)-O(3)	61.73

Structure II

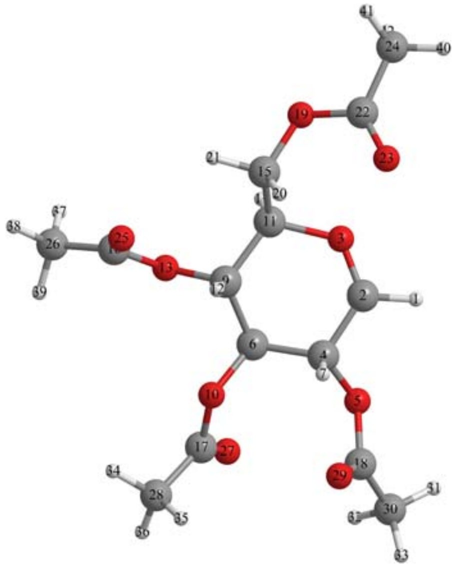

Bond	Bond Length (Å)	Bond Angle	Value (º)	Dihedral Angle	Value (º)
H(31)-C(30)	1.09	H(31)-C(30)-H(32)	107.80	H(42)-C(24)-C(22)-O(19)	-48.68
H(32)-C(30)	1.10	H(31)-C(30)-H(33)	110.72	H(42)-C(24)-C(22)-O(23)	131.85
H(33)-C(30)	1.09	H(31)-C(30)-C(18)	110.88	H(41)-C(24)-C(22)-O(19)	69.33
H(34)-C(28)	1.09	H(32)-C(30)-H(33)	109.74	H(41)-C(24)-C(22)-O(23)	-110.13

H(35)-C(28)	1.10	H(32)-C(30)-C(18)	108.16	H(40)-C(24)-C(22)-O(19)	-170.78
H(36)-C(28)	1.09	H(33)-C(30)-C(18)	109.49	H(40)-C(24)-C(22)-O(23)	9.75
H(37)-C(26)	1.09	H(34)-C(28)-H(35)	107.49	C(24)-C(22)-O(19)-C(15)	-172.70
H(38)-C(26)	1.09	H(34)-C(28)-H(36)	110.51	O(23)-C(22)-O(19)-C(15)	6.80
H(39)-C(26)	1.10	H(34)-C(28)-C(17)	110.87	H(33)-C(30)-C(18)-O(5)	168.19
H(40)-C(24)	1.09	H(35)-C(28)-H(36)	109.53	H(33)-C(30)-C(18)-O(29)	-13.59
H(41)-C(24)	1.10	H(35)-C(28)-C(17)	109.03	H(32)-C(30)-C(18)-O(5)	-72.28
H(42)-C(24)	1.09	H(36)-C(28)-C(17)	109.37	H(32)-C(30)-C(18)-O(29)	105.94
O(23)-C(22)	1.21	H(37)-C(26)-H(38)	110.17	H(31)-C(30)-C(18)-O(5)	45.72
C(24)-C(22)	1.50	H(37)-C(26)-H(39)	107.32	H(31)-C(30)-C(18)-O(29)	-136.06
C(22)-O(19)	1.38	H(37)-C(26)-C(16)	110.32	H(36)-C(28)-C(17)-O(10)	171.03
O(29)-C(18)	1.20	H(38)-C(26)-H(39)	109.88	H(36)-C(28)-C(17)-O(27)	-10.57
C(30)-C(18)	1.50	H(38)-C(26)-C(16)	109.29	H(35)-C(28)-C(17)-O(10)	-69.22
O(27)-C(17)	1.21	H(39)-C(26)-C(16)	109.85	H(35)-C(28)-C(17)-O(27)	109.17
C(28)-C(17)	1.50	H(40)-C(24)-H(41)	109.42	H(34)-C(28)-C(17)-O(10)	48.93
O(25)-C(16)	1.21	H(40)-C(24)-H(42)	110.53	H(34)-C(28)-C(17)-O(27)	-132.68
C(26)-C(16)	1.50	H(40)-C(24)-C(22)	109.51	H(39)-C(26)-C(16)-O(13)	63.08
O(19)-C(15)	1.42	H(41)-C(24)-H(42)	107.31	H(39)-C(26)-C(16)-O(25)	-117.41
H(20)-C(15)	1.09	H(41)-C(24)-C(22)	109.36	H(38)-C(26)-C(16)-O(13)	-176.30
H(21)-C(15)	1.09	H(42)-C(24)-C(22)	110.68	H(38)-C(26)-C(16)-O(25)	3.20
C(16)-O(13)	1.39	O(23)-C(22)-C(24)	127.10	H(37)-C(26)-C(16)-O(13)	-55.03
H(14)-C(11)	1.09	O(23)-C(22)-O(19)	122.18	H(37)-C(26)-C(16)-O(25)	124.48
C(15)-C(11)	1.53	C(24)-C(22)-O(19)	110.72	C(22)-O(19)-C(15)-C(11)	-95.71
C(17)-O(10)	1.39	C(22)-O(19)-C(15)	116.16	C(22)-O(19)-C(15)-H(21)	146.54
H(12)-C(9)	1.10	O(29)-C(18)-C(30)	128.63	C(22)-O(19)-C(15)-H(20)	27.58
O(13)-C(9)	1.43	O(29)-C(18)-O(5)	120.76	C(26)-C(16)-O(13)-C(9)	-173.35
C(11)-C(9)	1.54	C(30)-C(18)-O(5)	110.59	O(25)-C(16)-O(13)-C(9)	7.11
H(8)-C(6)	1.09	O(27)-C(17)-C(28)	127.57	H(21)-C(15)-C(11)-O(3)	178.96
O(10)-C(6)	1.42	O(27)-C(17)-O(10)	122.07	H(21)-C(15)-C(11)-C(9)	-57.02
C(9)-C(6)	1.53	C(28)-C(17)-O(10)	110.34	H(21)-C(15)-C(11)-H(14)	69.52
C(18)-O(5)	1.40	O(25)-C(16)-C(26)	127.65	H(20)-C(15)-C(11)-O(3)	-62.41
O(5)-C(4)	1.42	O(25)-C(16)-O(13)	121.88	H(20)-C(15)-C(11)-C(9)	61.61
H(7)-C(4)	1.11	C(26)-C(16)-O(13)	110.48	H(20)-C(15)-C(11)-H(14)	-171.85
C(6)-C(4)	1.54	O(19)-C(15)-H(20)	110.76	O(19)-C(15)-C(11)-O(3)	61.27
C(11)-O(3)	1.51	O(19)-C(15)-H(21)	107.37	O(19)-C(15)-C(11)-C(9)	-174.72
C(4)-C(2)	1.48	O(19)-C(15)-C(11)	111.99	O(19)-C(15)-C(11)-H(14)	-48.17
O(3)-C(2)	1.25	H(20)-C(15)-H(21)	109.04	C(28)-C(17)-O(10)-C(6)	-179.03
C(2)-H(1)	1.09	H(20)-C(15)-C(11)	110.07	O(27)-C(17)-O(10)-C(6)	2.47
		H(21)-C(15)-C(11)	107.47	C(16)-O(13)-C(9)-C(6)	136.02
		C(16)-O(13)-C(9)	116.72	C(16)-O(13)-C(9)-C(11)	-100.92
		H(14)-C(11)-C(15)	111.36	C(16)-O(13)-C(9)-H(12)	17.90
		H(14)-C(11)-C(9)	111.23	O(3)-C(11)-C(9)-C(6)	-29.95
		H(14)-C(11)-O(3)	101.60	O(3)-C(11)-C(9)-O(13)	-148.40
		C(15)-C(11)-C(9)	113.61	O(3)-C(11)-C(9)-H(12)	93.15
		C(15)-C(11)-O(3)	105.53	C(15)-C(11)-C(9)-C(6)	-149.96
		C(9)-C(11)-O(3)	112.74	C(15)-C(11)-C(9)-O(13)	91.60
		C(17)-O(10)-C(6)	117.40	C(15)-C(11)-C(9)-H(12)	-26.85
		H(12)-C(9)-O(13)	109.15	H(14)-C(11)-C(9)-C(6)	83.43
		H(12)-C(9)-C(11)	109.73	H(14)-C(11)-C(9)-O(13)	-35.02

		H(12)-C(9)-C(6)	109.26	H(14)-C(11)-C(9)-H(12)	-153.47
		O(13)-C(9)-C(11)	107.27	C(17)-O(10)-C(6)-C(4)	-93.52
		O(13)-C(9)-C(6)	107.00	C(17)-O(10)-C(6)-C(9)	147.42
		C(11)-C(9)-C(6)	114.29	C(17)-O(10)-C(6)-H(8)	26.76
		H(8)-C(6)-O(10)	110.14	C(11)-C(9)-C(6)-C(4)	51.37
		H(8)-C(6)-C(9)	111.65	C(11)-C(9)-C(6)-O(10)	170.48
		H(8)-C(6)-C(4)	108.94	C(11)-C(9)-C(6)-H(8)	-69.85
		O(10)-C(6)-C(9)	105.56	O(13)-C(9)-C(6)-C(4)	169.96
		O(10)-C(6)-C(4)	110.31	O(13)-C(9)-C(6)-O(10)	-70.93
		C(9)-C(6)-C(4)	110.23	O(13)-C(9)-C(6)-H(8)	48.74
		C(18)-O(5)-C(4)	114.75	H(12)-C(9)-C(6)-C(4)	-71.99
		O(5)-C(4)-H(7)	108.86	H(12)-C(9)-C(6)-O(10)	47.12
		O(5)-C(4)-C(6)	113.26	H(12)-C(9)-C(6)-H(8)	166.79
		O(5)-C(4)-C(2)	104.38	C(30)-C(18)-O(5)-C(4)	173.18
		H(7)-C(4)-C(6)	108.94	O(29)-C(18)-O(5)-C(4)	-5.20
		H(7)-C(4)-C(2)	109.10	C(18)-O(5)-C(4)-C(2)	138.86
		C(6)-C(4)-C(2)	112.15	C(18)-O(5)-C(4)-C(6)	-98.87
		C(11)-O(3)-C(2)	124.59	C(18)-O(5)-C(4)-H(7)	22.47
		C(4)-C(2)-O(3)	125.45	C(9)-C(6)-C(4)-C(2)	-45.00
		C(4)-C(2)-H(1)	119.92	C(9)-C(6)-C(4)-H(7)	75.87
		O(3)-C(2)-H(1)	114.58	C(9)-C(6)-C(4)-O(5)	-162.83
				O(10)-C(6)-C(4)-C(2)	-161.17
				O(10)-C(6)-C(4)-H(7)	-40.30
				O(10)-C(6)-C(4)-O(5)	80.99
				H(8)-C(6)-C(4)-C(2)	77.83
				H(8)-C(6)-C(4)-H(7)	-161.30
				H(8)-C(6)-C(4)-O(5)	-40.01
				C(9)-C(11)-O(3)-C(2)	1.58
				C(15)-C(11)-O(3)-C(2)	126.14
				H(14)-C(11)-O(3)-C(2)	-117.56
				C(6)-C(4)-C(2)-H(1)	-158.09
				C(6)-C(4)-C(2)-O(3)	19.13
				H(7)-C(4)-C(2)-H(1)	81.14
				H(7)-C(4)-C(2)-O(3)	-101.64
				O(5)-C(4)-C(2)-H(1)	-35.09
				O(5)-C(4)-C(2)-O(3)	142.13
				C(11)-O(3)-C(2)-H(1)	-178.89
				C(11)-O(3)-C(2)-C(4)	3.76

Structure III-a

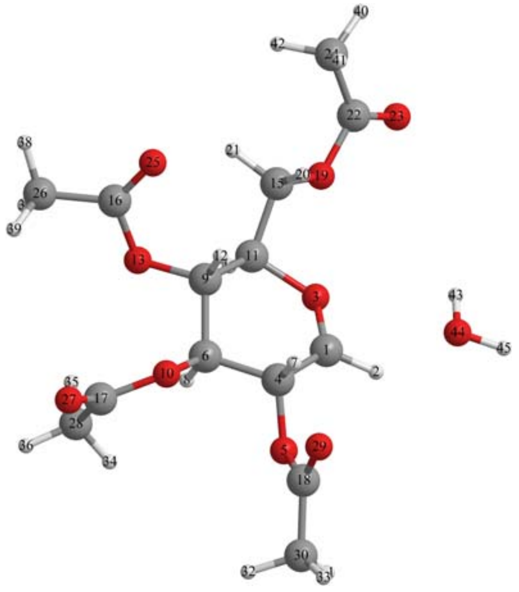

Bond	Bond Length (Å)	Bond Angle	Value (º)	Dihedral Angle	Value (º)
H(45)-O(44)	0.97	H(45)-O(44)-H(43)	104.78	H(42)-C(24)-C(22)-O(19)	-58.77
O(44)-H(43)	0.97	H(31)-C(30)-H(32)	107.63	H(42)-C(24)-C(22)-O(23)	120.34
H(31)-C(30)	1.09	H(31)-C(30)-H(33)	110.53	H(41)-C(24)-C(22)-O(19)	61.56
H(32)-C(30)	1.10	H(31)-C(30)-C(18)	110.54	H(41)-C(24)-C(22)-O(23)	-119.33
H(33)-C(30)	1.09	H(32)-C(30)-H(33)	109.45	H(40)-C(24)-C(22)-O(19)	-178.55
H(34)-C(28)	1.10	H(32)-C(30)-C(18)	109.40	H(40)-C(24)-C(22)-O(23)	0.56
H(35)-C(28)	1.09	H(33)-C(30)-C(18)	109.25	C(24)-C(22)-O(19)-C(15)	-10.67
H(36)-C(28)	1.09	H(34)-C(28)-H(35)	108.42	O(23)-C(22)-O(19)-C(15)	170.14
H(37)-C(26)	1.09	H(34)-C(28)-H(36)	109.00	H(33)-C(30)-C(18)-O(5)	171.27
H(38)-C(26)	1.09	H(34)-C(28)-C(17)	111.66	H(33)-C(30)-C(18)-O(29)	-8.81
H(39)-C(26)	1.10	H(35)-C(28)-H(36)	108.87	H(32)-C(30)-C(18)-O(5)	-68.93
H(40)-C(24)	1.09	H(35)-C(28)-C(17)	111.34	H(32)-C(30)-C(18)-O(29)	110.99
H(41)-C(24)	1.10	H(36)-C(28)-C(17)	107.49	H(31)-C(30)-C(18)-O(5)	49.42
H(42)-C(24)	1.10	H(37)-C(26)-H(38)	110.52	H(31)-C(30)-C(18)-O(29)	-130.65
O(23)-C(22)	1.20	H(37)-C(26)-H(39)	107.73	H(36)-C(28)-C(17)-O(10)	174.02
C(24)-C(22)	1.51	H(37)-C(26)-C(16)	110.94	H(36)-C(28)-C(17)-O(27)	-4.07
C(22)-O(19)	1.39	H(38)-C(26)-H(39)	109.17	H(35)-C(28)-C(17)-O(10)	-66.83
O(29)-C(18)	1.20	H(38)-C(26)-C(16)	109.34	H(35)-C(28)-C(17)-O(27)	115.09
C(30)-C(18)	1.50	H(39)-C(26)-C(16)	109.11	H(34)-C(28)-C(17)-O(10)	54.52
O(27)-C(17)	1.20	H(40)-C(24)-H(41)	108.98	H(34)-C(28)-C(17)-O(27)	-123.57
C(28)-C(17)	1.51	H(40)-C(24)-H(42)	109.10	H(39)-C(26)-C(16)-O(13)	72.68
O(25)-C(16)	1.21	H(40)-C(24)-C(22)	108.17	H(39)-C(26)-C(16)-O(25)	-106.94
C(26)-C(16)	1.50	H(41)-C(24)-H(42)	107.72	H(38)-C(26)-C(16)-O(13)	-167.99
O(19)-C(15)	1.42	H(41)-C(24)-C(22)	111.64	H(38)-C(26)-C(16)-O(25)	12.38
H(20)-C(15)	1.10	H(42)-C(24)-C(22)	111.19	H(37)-C(26)-C(16)-O(13)	-45.85
H(21)-C(15)	1.09	O(23)-C(22)-C(24)	125.78	H(37)-C(26)-C(16)-O(25)	134.52
C(16)-O(13)	1.39	O(23)-C(22)-O(19)	116.75	C(22)-O(19)-C(15)-C(11)	179.83
H(14)-C(11)	1.09	C(24)-C(22)-O(19)	117.46	C(22)-O(19)-C(15)-H(21)	61.70
C(15)-C(11)	1.52	C(22)-O(19)-C(15)	122.24	C(22)-O(19)-C(15)-H(20)	-60.51
C(17)-O(10)	1.40	O(29)-C(18)-C(30)	128.32	C(26)-C(16)-O(13)-C(9)	-168.87

H(12)-C(9)	1.09	O(29)-C(18)-O(5)	121.02	O(25)-C(16)-O(13)-C(9)	10.79
O(13)-C(9)	1.43	C(30)-C(18)-O(5)	110.66	H(21)-C(15)-C(11)-O(3)	174.98
C(11)-C(9)	1.54	O(27)-C(17)-C(28)	125.30	H(21)-C(15)-C(11)-C(9)	-61.69
H(8)-C(6)	1.09	O(27)-C(17)-O(10)	116.50	H(21)-C(15)-C(11)-H(14)	66.15
O(10)-C(6)	1.41	C(28)-C(17)-O(10)	118.17	H(20)-C(15)-C(11)-O(3)	-67.42
C(9)-C(6)	1.54	O(25)-C(16)-C(26)	127.76	H(20)-C(15)-C(11)-C(9)	55.91
C(18)-O(5)	1.40	O(25)-C(16)-O(13)	121.44	H(20)-C(15)-C(11)-H(14)	-176.26
O(5)-C(4)	1.42	C(26)-C(16)-O(13)	110.80	O(19)-C(15)-C(11)-O(3)	53.57
H(7)-C(4)	1.11	O(19)-C(15)-H(20)	112.01	O(19)-C(15)-C(11)-C(9)	176.91
C(6)-C(4)	1.54	O(19)-C(15)-H(21)	113.16	O(19)-C(15)-C(11)-H(14)	-55.26
C(11)-O(3)	1.51	O(19)-C(15)-C(11)	105.55	C(28)-C(17)-O(10)-C(6)	15.26
H(2)-C(1)	1.10	H(20)-C(15)-H(21)	107.88	O(27)-C(17)-O(10)-C(6)	-166.48
C(4)-C(1)	1.48	H(20)-C(15)-C(11)	109.97	C(16)-O(13)-C(9)-C(6)	141.90
O(3)-C(1)	1.26	H(21)-C(15)-C(11)	108.19	C(16)-O(13)-C(9)-C(11)	-97.01
		C(16)-O(13)-C(9)	116.41	C(16)-O(13)-C(9)-H(12)	23.65
		H(14)-C(11)-C(15)	110.18	O(3)-C(11)-C(9)-C(6)	-38.31
		H(14)-C(11)-C(9)	111.25	O(3)-C(11)-C(9)-O(13)	-156.09
		H(14)-C(11)-O(3)	102.03	O(3)-C(11)-C(9)-H(12)	83.95
		C(15)-C(11)-C(9)	116.38	C(15)-C(11)-C(9)-C(6)	-157.79
		C(15)-C(11)-O(3)	104.28	C(15)-C(11)-C(9)-O(13)	84.43
		C(9)-C(11)-O(3)	111.56	C(15)-C(11)-C(9)-H(12)	-35.53
		C(17)-O(10)-C(6)	123.35	H(14)-C(11)-C(9)-C(6)	74.90
		H(12)-C(9)-O(13)	109.28	H(14)-C(11)-C(9)-O(13)	-42.88
		H(12)-C(9)-C(11)	110.42	H(14)-C(11)-C(9)-H(12)	-162.84
		H(12)-C(9)-C(6)	109.45	C(17)-O(10)-C(6)-C(4)	-140.19
		O(13)-C(9)-C(11)	108.85	C(17)-O(10)-C(6)-C(9)	102.09
		O(13)-C(9)-C(6)	106.72	C(17)-O(10)-C(6)-H(8)	-20.38
		C(11)-C(9)-C(6)	112.02	C(11)-C(9)-C(6)-C(4)	58.11
		H(8)-C(6)-O(10)	112.26	C(11)-C(9)-C(6)-O(10)	175.03
		H(8)-C(6)-C(9)	110.15	C(11)-C(9)-C(6)-H(8)	-61.24
		H(8)-C(6)-C(4)	109.00	O(13)-C(9)-C(6)-C(4)	177.15
		O(10)-C(6)-C(9)	109.22	O(13)-C(9)-C(6)-O(10)	-65.93
		O(10)-C(6)-C(4)	107.43	O(13)-C(9)-C(6)-H(8)	57.80
		C(9)-C(6)-C(4)	108.69	H(12)-C(9)-C(6)-C(4)	-64.71
		C(18)-O(5)-C(4)	114.42	H(12)-C(9)-C(6)-O(10)	52.21
		O(5)-C(4)-H(7)	107.83	H(12)-C(9)-C(6)-H(8)	175.94
		O(5)-C(4)-C(6)	111.94	C(30)-C(18)-O(5)-C(4)	173.86
		O(5)-C(4)-C(1)	108.54	O(29)-C(18)-O(5)-C(4)	-6.07
		H(7)-C(4)-C(6)	109.39	C(18)-O(5)-C(4)-C(1)	119.21
		H(7)-C(4)-C(1)	106.95	C(18)-O(5)-C(4)-C(6)	-116.68
		C(6)-C(4)-C(1)	111.99	C(18)-O(5)-C(4)-H(7)	3.69
		C(11)-O(3)-C(1)	125.27	C(9)-C(6)-C(4)-C(1)	-48.76
		H(2)-C(1)-C(4)	123.80	C(9)-C(6)-C(4)-H(7)	69.62
		H(2)-C(1)-O(3)	112.10	C(9)-C(6)-C(4)-O(5)	-170.92
		C(4)-C(1)-O(3)	124.10	O(10)-C(6)-C(4)-C(1)	-166.82
				O(10)-C(6)-C(4)-H(7)	-48.43
				O(10)-C(6)-C(4)-O(5)	71.02
				H(8)-C(6)-C(4)-C(1)	71.31
				H(8)-C(6)-C(4)-H(7)	-170.31

				H(8)-C(6)-C(4)-O(5)	-50.85
				C(9)-C(11)-O(3)-C(1)	9.78
				C(15)-C(11)-O(3)-C(1)	136.20
				H(14)-C(11)-O(3)-C(1)	-109.08
				C(6)-C(4)-C(1)-O(3)	22.07
				C(6)-C(4)-C(1)-H(2)	-158.76
				H(7)-C(4)-C(1)-O(3)	-97.75
				H(7)-C(4)-C(1)-H(2)	81.42
				O(5)-C(4)-C(1)-O(3)	146.16
				O(5)-C(4)-C(1)-H(2)	-34.68
				C(11)-O(3)-C(1)-C(4)	-1.59
				C(11)-O(3)-C(1)-H(2)	179.16

Structure III-b

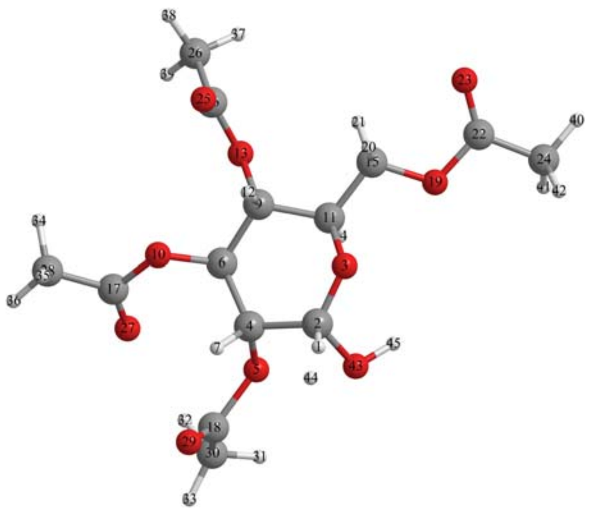

Bond	Bond Length (Å)	Bond Angle	Value (°)	Dihedral Angle	Value (°)
H(45)-O(43)	0.98	H(31)-C(30)-H(32)	108.33	H(42)-C(24)-C(22)-O(19)	-64.23
H(31)-C(30)	1.09	H(31)-C(30)-H(33)	110.49	H(42)-C(24)-C(22)-O(23)	115.43
H(32)-C(30)	1.10	H(31)-C(30)-C(18)	111.59	H(41)-C(24)-C(22)-O(19)	54.51
H(33)-C(30)	1.09	H(32)-C(30)-H(33)	109.95	H(41)-C(24)-C(22)-O(23)	-125.83
H(34)-C(28)	1.10	H(32)-C(30)-C(18)	107.39	H(40)-C(24)-C(22)-O(19)	175.61
H(35)-C(28)	1.09	H(33)-C(30)-C(18)	109.04	H(40)-C(24)-C(22)-O(23)	-4.72
H(36)-C(28)	1.09	H(34)-C(28)-H(35)	107.16	C(24)-C(22)-O(19)-C(15)	178.48
H(37)-C(26)	1.10	H(34)-C(28)-H(36)	109.90	O(23)-C(22)-O(19)-C(15)	-1.20
H(38)-C(26)	1.09	H(34)-C(28)-C(17)	109.87	H(33)-C(30)-C(18)-O(5)	169.36
H(39)-C(26)	1.09	H(35)-C(28)-H(36)	110.19	H(33)-C(30)-C(18)-O(29)	-12.09
H(40)-C(24)	1.09	H(35)-C(28)-C(17)	110.16	H(32)-C(30)-C(18)-O(5)	-71.55
H(41)-C(24)	1.09	H(36)-C(28)-C(17)	109.53	H(32)-C(30)-C(18)-O(29)	107.00
H(42)-C(24)	1.10	H(37)-C(26)-H(38)	109.49	H(31)-C(30)-C(18)-O(5)	47.03
O(23)-C(22)	1.21	H(37)-C(26)-H(39)	107.41	H(31)-C(30)-C(18)-O(29)	-134.42
C(24)-C(22)	1.51	H(37)-C(26)-C(16)	109.81	H(36)-C(28)-C(17)-O(10)	-176.26
C(22)-O(19)	1.38	H(38)-C(26)-H(39)	110.32	H(36)-C(28)-C(17)-O(27)	3.49
O(29)-C(18)	1.18	H(38)-C(26)-C(16)	109.27	H(35)-C(28)-C(17)-O(10)	-54.90
C(30)-C(18)	1.48	H(39)-C(26)-C(16)	110.52	H(35)-C(28)-C(17)-O(27)	124.85

O(27)-C(17)	1.21	H(40)-C(24)-H(41)	109.93	H(34)-C(28)-C(17)-O(10)	62.93
C(28)-C(17)	1.50	H(40)-C(24)-H(42)	109.50	H(34)-C(28)-C(17)-O(27)	-117.32
O(25)-C(16)	1.20	H(40)-C(24)-C(22)	109.21	H(39)-C(26)-C(16)-O(13)	53.15
C(26)-C(16)	1.50	H(41)-C(24)-H(42)	107.65	H(39)-C(26)-C(16)-O(25)	-127.66
O(19)-C(15)	1.43	H(41)-C(24)-C(22)	110.59	H(38)-C(26)-C(16)-O(13)	174.74
H(20)-C(15)	1.10	H(42)-C(24)-C(22)	109.93	H(38)-C(26)-C(16)-O(25)	-6.08
H(21)-C(15)	1.09	O(23)-C(22)-C(24)	126.88	H(37)-C(26)-C(16)-O(13)	-65.16
C(16)-O(13)	1.39	O(23)-C(22)-O(19)	122.07	H(37)-C(26)-C(16)-O(25)	114.02
H(14)-C(11)	1.10	C(24)-C(22)-O(19)	111.05	C(22)-O(19)-C(15)-C(11)	169.91
C(15)-C(11)	1.51	C(22)-O(19)-C(15)	114.35	C(22)-O(19)-C(15)-H(21)	50.45
C(17)-O(10)	1.37	O(29)-C(18)-C(30)	133.74	C(22)-O(19)-C(15)-H(20)	-69.53
H(12)-C(9)	1.09	O(29)-C(18)-O(5)	117.76	C(26)-C(16)-O(13)-C(9)	-179.20
O(13)-C(9)	1.42	C(30)-C(18)-O(5)	108.49	O(25)-C(16)-O(13)-C(9)	1.57
C(11)-C(9)	1.53	O(27)-C(17)-C(28)	126.79	H(21)-C(15)-C(11)-O(3)	-172.78
H(8)-C(6)	1.09	O(27)-C(17)-O(10)	121.87	H(21)-C(15)-C(11)-C(9)	-55.20
O(10)-C(6)	1.43	C(28)-C(17)-O(10)	111.34	H(21)-C(15)-C(11)-H(14)	69.31
C(9)-C(6)	1.53	O(25)-C(16)-C(26)	127.30	H(20)-C(15)-C(11)-O(3)	-52.77
C(18)-O(5)	1.53	O(25)-C(16)-O(13)	122.53	H(20)-C(15)-C(11)-C(9)	64.81
O(5)-C(4)	1.47	C(26)-C(16)-O(13)	110.16	H(20)-C(15)-C(11)-H(14)	-170.68
H(7)-C(4)	1.09	O(19)-C(15)-H(20)	110.50	O(19)-C(15)-C(11)-O(3)	67.54
C(6)-C(4)	1.55	O(19)-C(15)-H(21)	110.37	O(19)-C(15)-C(11)-C(9)	-174.88
C(11)-O(3)	1.46	O(19)-C(15)-C(11)	106.58	O(19)-C(15)-C(11)-H(14)	-50.37
O(43)-C(2)	1.50	H(20)-C(15)-H(21)	108.48	C(28)-C(17)-O(10)-C(6)	-173.32
C(4)-C(2)	1.58	H(20)-C(15)-C(11)	110.89	O(27)-C(17)-O(10)-C(6)	6.92
O(3)-C(2)	1.36	H(21)-C(15)-C(11)	110.04	C(16)-O(13)-C(9)-C(6)	118.61
C(2)-H(1)	1.09	C(16)-O(13)-C(9)	116.67	C(16)-O(13)-C(9)-C(11)	-123.43
		H(14)-C(11)-C(15)	108.53	C(16)-O(13)-C(9)-H(12)	-2.49
		H(14)-C(11)-C(9)	110.70	O(3)-C(11)-C(9)-C(6)	-68.20
		H(14)-C(11)-O(3)	109.47	O(3)-C(11)-C(9)-O(13)	174.43
		C(15)-C(11)-C(9)	114.90	O(3)-C(11)-C(9)-H(12)	53.67
		C(15)-C(11)-O(3)	106.76	C(15)-C(11)-C(9)-C(6)	173.96
		C(9)-C(11)-O(3)	106.30	C(15)-C(11)-C(9)-O(13)	56.58
		C(17)-O(10)-C(6)	116.38	C(15)-C(11)-C(9)-H(12)	-64.17
		H(12)-C(9)-O(13)	109.56	H(14)-C(11)-C(9)-C(6)	50.59
		H(12)-C(9)-C(11)	109.87	H(14)-C(11)-C(9)-O(13)	-66.78
		H(12)-C(9)-C(6)	111.31	H(14)-C(11)-C(9)-H(12)	172.46
		O(13)-C(9)-C(11)	110.11	C(17)-O(10)-C(6)-C(4)	-77.38
		O(13)-C(9)-C(6)	107.51	C(17)-O(10)-C(6)-C(9)	157.52
		C(11)-C(9)-C(6)	108.45	C(17)-O(10)-C(6)-H(8)	40.56
		H(8)-C(6)-O(10)	109.02	C(11)-C(9)-C(6)-C(4)	43.89
		H(8)-C(6)-C(9)	108.23	C(11)-C(9)-C(6)-O(10)	167.30
		H(8)-C(6)-C(4)	107.20	C(11)-C(9)-C(6)-H(8)	-75.23
		O(10)-C(6)-C(9)	107.22	O(13)-C(9)-C(6)-C(4)	162.92
		O(10)-C(6)-C(4)	111.14	O(13)-C(9)-C(6)-O(10)	-73.67
		C(9)-C(6)-C(4)	113.92	O(13)-C(9)-C(6)-H(8)	43.80
		C(18)-O(5)-C(4)	117.05	H(12)-C(9)-C(6)-C(4)	-77.09
		H(45)-O(43)-C(2)	109.36	H(12)-C(9)-C(6)-O(10)	46.32
		O(5)-C(4)-H(7)	107.57	H(12)-C(9)-C(6)-H(8)	163.79
		O(5)-C(4)-C(6)	108.28	C(30)-C(18)-O(5)-C(4)	161.93

		O(5)-C(4)-C(2)	104.41	O(29)-C(18)-O(5)-C(4)	-16.88
		H(7)-C(4)-C(6)	110.24	C(18)-O(5)-C(4)-C(2)	122.03
		H(7)-C(4)-C(2)	108.78	C(18)-O(5)-C(4)-C(6)	-112.57
		C(6)-C(4)-C(2)	117.04	C(18)-O(5)-C(4)-H(7)	6.56
		C(11)-O(3)-C(2)	118.04	C(9)-C(6)-C(4)-C(2)	-12.46
		O(43)-C(2)-C(4)	103.31	C(9)-C(6)-C(4)-H(7)	112.53
		O(43)-C(2)-O(3)	111.29	C(9)-C(6)-C(4)-O(5)	-130.04
		O(43)-C(2)-H(1)	104.56	O(10)-C(6)-C(4)-C(2)	-133.72
		C(4)-C(2)-O(3)	117.06	O(10)-C(6)-C(4)-H(7)	-8.72
		C(4)-C(2)-H(1)	111.74	O(10)-C(6)-C(4)-O(5)	108.70
		O(3)-C(2)-H(1)	108.13	H(8)-C(6)-C(4)-C(2)	107.24
				H(8)-C(6)-C(4)-H(7)	-127.76
				H(8)-C(6)-C(4)-O(5)	-10.33
				C(9)-C(11)-O(3)-C(2)	64.05
				C(15)-C(11)-O(3)-C(2)	-172.85
				H(14)-C(11)-O(3)-C(2)	-55.55
				H(45)-O(43)-C(2)-H(1)	-115.47
				H(45)-O(43)-C(2)-O(3)	1.04
				H(45)-O(43)-C(2)-C(4)	127.48
				C(6)-C(4)-C(2)-H(1)	129.59
				C(6)-C(4)-C(2)-O(3)	4.14
				C(6)-C(4)-C(2)-O(43)	-118.54
				H(7)-C(4)-C(2)-H(1)	3.86
				H(7)-C(4)-C(2)-O(3)	-121.59
				H(7)-C(4)-C(2)-O(43)	115.73
				O(5)-C(4)-C(2)-H(1)	-110.76
				O(5)-C(4)-C(2)-O(3)	123.79
				O(5)-C(4)-C(2)-O(43)	1.11
				C(11)-O(3)-C(2)-H(1)	-158.31
				C(11)-O(3)-C(2)-C(4)	-31.07
				C(11)-O(3)-C(2)-O(43)	87.39

Structure IV-a

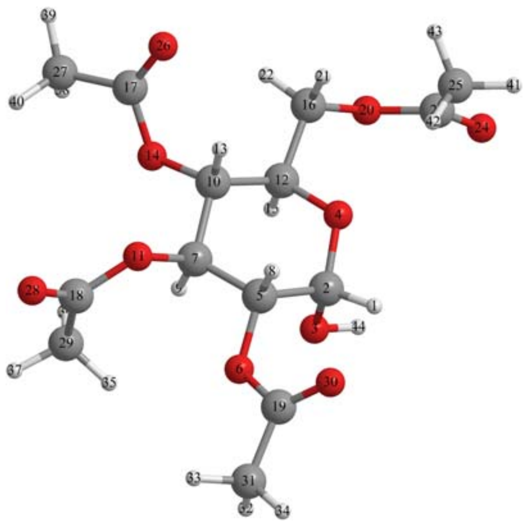

Bond	Bond Length (Å)	Bond Angle	Value (º)	Dihedral Angle	Value (º)
H(32)-C(31)	1.09	H(32)-C(31)-H(33)	107.54	H(43)-C(25)-C(23)-O(20)	-55.72
H(33)-C(31)	1.10	H(32)-C(31)-H(34)	110.08	H(43)-C(25)-C(23)-O(24)	121.72
H(34)-C(31)	1.09	H(32)-C(31)-C(19)	110.17	H(42)-C(25)-C(23)-O(20)	64.92
H(35)-C(29)	1.09	H(33)-C(31)-H(34)	109.75	H(42)-C(25)-C(23)-O(24)	-117.64
H(36)-C(29)	1.10	H(33)-C(31)-C(19)	109.91	H(41)-C(25)-C(23)-O(20)	-175.26
H(37)-C(29)	1.09	H(34)-C(31)-C(19)	109.37	H(41)-C(25)-C(23)-O(24)	2.18
H(38)-C(27)	1.09	H(35)-C(29)-H(36)	108.07	C(25)-C(23)-O(20)-C(16)	-15.91
H(39)-C(27)	1.09	H(35)-C(29)-H(37)	109.58	O(24)-C(23)-O(20)-C(16)	166.51
H(40)-C(27)	1.10	H(35)-C(29)-C(18)	111.34	H(34)-C(31)-C(19)-O(6)	176.93
H(41)-C(25)	1.09	H(36)-C(29)-H(37)	109.05	H(34)-C(31)-C(19)-O(30)	-3.18
H(42)-C(25)	1.09	H(36)-C(29)-C(18)	111.28	H(33)-C(31)-C(19)-O(6)	-62.52
H(43)-C(25)	1.10	H(37)-C(29)-C(18)	107.50	H(33)-C(31)-C(19)-O(30)	117.37
O(24)-C(23)	1.21	H(38)-C(27)-H(39)	110.42	H(32)-C(31)-C(19)-O(6)	55.81
C(25)-C(23)	1.52	H(38)-C(27)-H(40)	107.59	H(32)-C(31)-C(19)-O(30)	-124.30
C(23)-O(20)	1.37	H(38)-C(27)-C(17)	110.71	H(37)-C(29)-C(18)-O(11)	179.23
O(30)-C(19)	1.21	H(39)-C(27)-H(40)	109.32	H(37)-C(29)-C(18)-O(28)	-1.31
C(31)-C(19)	1.51	H(39)-C(27)-C(17)	109.43	H(36)-C(29)-C(18)-O(11)	-61.42
O(28)-C(18)	1.21	H(40)-C(27)-C(17)	109.33	H(36)-C(29)-C(18)-O(28)	118.04
C(29)-C(18)	1.52	H(41)-C(25)-H(42)	109.51	H(35)-C(29)-C(18)-O(11)	59.21
O(26)-C(17)	1.21	H(41)-C(25)-H(43)	109.11	H(35)-C(29)-C(18)-O(28)	-121.33
C(27)-C(17)	1.51	H(41)-C(25)-C(23)	107.59	H(40)-C(27)-C(17)-O(14)	71.20
O(20)-C(16)	1.43	H(42)-C(25)-H(43)	108.18	H(40)-C(27)-C(17)-O(26)	-108.63
H(21)-C(16)	1.09	H(42)-C(25)-C(23)	111.05	H(39)-C(27)-C(17)-O(14)	-169.09
H(22)-C(16)	1.09	H(43)-C(25)-C(23)	111.38	H(39)-C(27)-C(17)-O(26)	11.07
C(17)-O(14)	1.37	O(24)-C(23)-C(25)	123.50	H(38)-C(27)-C(17)-O(14)	-47.15
H(15)-C(12)	1.10	O(24)-C(23)-O(20)	117.81	H(38)-C(27)-C(17)-O(26)	133.01
C(16)-C(12)	1.53	C(25)-C(23)-O(20)	118.64	C(23)-O(20)-C(16)-C(12)	-95.26
C(18)-O(11)	1.37	C(23)-O(20)-C(16)	122.95	C(23)-O(20)-C(16)-H(22)	146.20
H(13)-C(10)	1.09	O(30)-C(19)-C(31)	126.12	C(23)-O(20)-C(16)-H(21)	28.16
O(14)-C(10)	1.44	O(30)-C(19)-O(6)	123.19	C(27)-C(17)-O(14)-C(10)	-171.81
C(12)-C(10)	1.54	C(31)-C(19)-O(6)	110.69	O(26)-C(17)-O(14)-C(10)	8.03
H(9)-C(7)	1.09	O(28)-C(18)-C(29)	123.54	H(22)-C(16)-C(12)-O(4)	-177.17
O(11)-C(7)	1.43	O(28)-C(18)-O(11)	117.71	H(22)-C(16)-C(12)-C(10)	-57.23
C(10)-C(7)	1.53	C(29)-C(18)-O(11)	118.74	H(22)-C(16)-C(12)-H(15)	64.96
C(19)-O(6)	1.36	O(26)-C(17)-C(27)	125.98	H(21)-C(16)-C(12)-O(4)	-57.62
O(6)-C(5)	1.44	O(26)-C(17)-O(14)	123.45	H(21)-C(16)-C(12)-C(10)	62.32
H(8)-C(5)	1.09	C(27)-C(17)-O(14)	110.57	H(21)-C(16)-C(12)-H(15)	-175.50
C(7)-C(5)	1.53	O(20)-C(16)-H(21)	112.54	O(20)-C(16)-C(12)-O(4)	66.85
C(12)-O(4)	1.43	O(20)-C(16)-H(22)	106.01	O(20)-C(16)-C(12)-C(10)	-173.21
H(44)-O(3)	0.97	O(20)-C(16)-C(12)	109.19	O(20)-C(16)-C(12)-H(15)	-51.02
O(3)-C(2)	1.40	H(21)-C(16)-H(22)	108.16	C(29)-C(18)-O(11)-C(7)	-2.33
C(5)-C(2)	1.54	H(21)-C(16)-C(12)	110.75	O(28)-C(18)-O(11)-C(7)	178.18
O(4)-C(2)	1.42	H(22)-C(16)-C(12)	110.06	C(17)-O(14)-C(10)-C(7)	138.56
C(2)-H(1)	1.09	C(17)-O(14)-C(10)	117.30	C(17)-O(14)-C(10)-C(12)	-101.74
		H(15)-C(12)-C(16)	108.46	C(17)-O(14)-C(10)-H(13)	19.99
		H(15)-C(12)-C(10)	109.30	O(4)-C(12)-C(10)-C(7)	-55.64
		H(15)-C(12)-O(4)	109.61	O(4)-C(12)-C(10)-O(14)	-172.76

		C(16)-C(12)-C(10)	114.27	O(4)-C(12)-C(10)-H(13)	66.24
		C(16)-C(12)-O(4)	106.46	C(16)-C(12)-C(10)-C(7)	-174.34
		C(10)-C(12)-O(4)	108.65	C(16)-C(12)-C(10)-O(14)	68.54
		C(18)-O(11)-C(7)	123.57	C(16)-C(12)-C(10)-H(13)	-52.46
		H(13)-C(10)-O(14)	109.07	H(15)-C(12)-C(10)-C(7)	63.93
		H(13)-C(10)-C(12)	110.32	H(15)-C(12)-C(10)-O(14)	-53.19
		H(13)-C(10)-C(7)	110.14	H(15)-C(12)-C(10)-H(13)	-174.19
		O(14)-C(10)-C(12)	110.96	C(18)-O(11)-C(7)-C(5)	-109.99
		O(14)-C(10)-C(7)	105.99	C(18)-O(11)-C(7)-C(10)	129.72
		C(12)-C(10)-C(7)	110.26	C(18)-O(11)-C(7)-H(9)	10.51
		H(9)-C(7)-O(11)	111.51	C(12)-C(10)-C(7)-C(5)	52.29
		H(9)-C(7)-C(10)	108.77	C(12)-C(10)-C(7)-O(11)	171.41
		H(9)-C(7)-C(5)	109.09	C(12)-C(10)-C(7)-H(9)	-67.65
		O(11)-C(7)-C(10)	107.61	O(14)-C(10)-C(7)-C(5)	172.45
		O(11)-C(7)-C(5)	109.01	O(14)-C(10)-C(7)-O(11)	-68.42
		C(10)-C(7)-C(5)	110.86	O(14)-C(10)-C(7)-H(9)	52.51
		C(19)-O(6)-C(5)	116.60	H(13)-C(10)-C(7)-C(5)	-69.70
		O(6)-C(5)-H(8)	109.16	H(13)-C(10)-C(7)-O(11)	49.43
		O(6)-C(5)-C(7)	106.41	H(13)-C(10)-C(7)-H(9)	170.37
		O(6)-C(5)-C(2)	112.49	C(31)-C(19)-O(6)-C(5)	176.64
		H(8)-C(5)-C(7)	110.07	O(30)-C(19)-O(6)-C(5)	-3.25
		H(8)-C(5)-C(2)	107.91	C(19)-O(6)-C(5)-C(2)	77.94
		C(7)-C(5)-C(2)	110.79	C(19)-O(6)-C(5)-C(7)	-160.56
		C(12)-O(4)-C(2)	114.69	C(19)-O(6)-C(5)-H(8)	-41.80
		H(44)-O(3)-C(2)	107.65	C(10)-C(7)-C(5)-C(2)	-51.98
		O(3)-C(2)-C(5)	108.11	C(10)-C(7)-C(5)-H(8)	67.29
		O(3)-C(2)-O(4)	112.80	C(10)-C(7)-C(5)-O(6)	-174.55
		O(3)-C(2)-H(1)	112.05	O(11)-C(7)-C(5)-C(2)	-170.25
		C(5)-C(2)-O(4)	108.80	O(11)-C(7)-C(5)-H(8)	-50.99
		C(5)-C(2)-H(1)	110.39	O(11)-C(7)-C(5)-O(6)	67.17
		O(4)-C(2)-H(1)	104.64	H(9)-C(7)-C(5)-C(2)	67.77
				H(9)-C(7)-C(5)-H(8)	-172.97
				H(9)-C(7)-C(5)-O(6)	-54.80
				C(10)-C(12)-O(4)-C(2)	63.17
				C(16)-C(12)-O(4)-C(2)	-173.32
				H(15)-C(12)-O(4)-C(2)	-56.20
				H(44)-O(3)-C(2)-H(1)	-54.16
				H(44)-O(3)-C(2)-O(4)	63.62
				H(44)-O(3)-C(2)-C(5)	-176.03
				C(7)-C(5)-C(2)-H(1)	169.37
				C(7)-C(5)-C(2)-O(4)	55.08
				C(7)-C(5)-C(2)-O(3)	-67.74
				H(8)-C(5)-C(2)-H(1)	48.81
				H(8)-C(5)-C(2)-O(4)	-65.48
				H(8)-C(5)-C(2)-O(3)	171.70
				O(6)-C(5)-C(2)-H(1)	-71.66
				O(6)-C(5)-C(2)-O(4)	174.05
				O(6)-C(5)-C(2)-O(3)	51.23
				C(12)-O(4)-C(2)-H(1)	179.37

				C(12)-O(4)-C(2)-C(5)	-62.64
				C(12)-O(4)-C(2)-O(3)	57.31

Structure IV-b

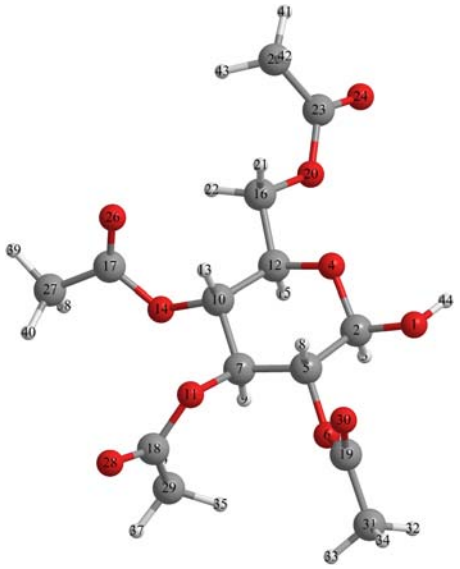

Bond	Bond Length (Å)	Bond Angle	Value (º)	Dihedral Angle	Value (º)
H(32)-C(31)	1.10	H(32)-C(31)-H(33)	107.53	H(43)-C(25)-C(23)-O(20)	-54.67
H(33)-C(31)	1.09	H(32)-C(31)-H(34)	109.85	H(43)-C(25)-C(23)-O(24)	125.98
H(34)-C(31)	1.09	H(32)-C(31)-C(19)	109.85	H(42)-C(25)-C(23)-O(20)	65.30
H(35)-C(29)	1.09	H(33)-C(31)-H(34)	109.91	H(42)-C(25)-C(23)-O(24)	-114.05
H(36)-C(29)	1.10	H(33)-C(31)-C(19)	110.37	H(41)-C(25)-C(23)-O(20)	-175.37
H(37)-C(29)	1.09	H(34)-C(31)-C(19)	109.31	H(41)-C(25)-C(23)-O(24)	5.29
H(38)-C(27)	1.09	H(35)-C(29)-H(36)	108.11	C(25)-C(23)-O(20)-C(16)	3.37
H(39)-C(27)	1.09	H(35)-C(29)-H(37)	109.49	O(24)-C(23)-O(20)-C(16)	-177.24
H(40)-C(27)	1.10	H(35)-C(29)-C(18)	111.47	H(34)-C(31)-C(19)-O(6)	-177.80
H(41)-C(25)	1.09	H(36)-C(29)-H(37)	109.05	H(34)-C(31)-C(19)-O(30)	2.12
H(42)-C(25)	1.10	H(36)-C(29)-C(18)	111.22	H(33)-C(31)-C(19)-O(6)	-56.81
H(43)-C(25)	1.10	H(37)-C(29)-C(18)	107.47	H(33)-C(31)-C(19)-O(30)	123.11
O(24)-C(23)	1.21	H(38)-C(27)-H(39)	110.47	H(32)-C(31)-C(19)-O(6)	61.60
C(25)-C(23)	1.52	H(38)-C(27)-H(40)	107.68	H(32)-C(31)-C(19)-O(30)	-118.48
C(23)-O(20)	1.37	H(38)-C(27)-C(17)	110.86	H(37)-C(29)-C(18)-O(11)	175.90
O(30)-C(19)	1.20	H(39)-C(27)-H(40)	109.15	H(37)-C(29)-C(18)-O(28)	-3.51
C(31)-C(19)	1.51	H(39)-C(27)-C(17)	109.43	H(36)-C(29)-C(18)-O(11)	-64.81
O(28)-C(18)	1.20	H(40)-C(27)-C(17)	109.21	H(36)-C(29)-C(18)-O(28)	115.78
C(29)-C(18)	1.52	H(41)-C(25)-H(42)	108.98	H(35)-C(29)-C(18)-O(11)	55.91
O(26)-C(17)	1.21	H(41)-C(25)-H(43)	109.50	H(35)-C(29)-C(18)-O(28)	-123.50
C(27)-C(17)	1.51	H(41)-C(25)-C(23)	108.03	H(40)-C(27)-C(17)-O(14)	73.37
O(20)-C(16)	1.43	H(42)-C(25)-H(43)	107.41	H(40)-C(27)-C(17)-O(26)	-106.37
H(21)-C(16)	1.09	H(42)-C(25)-C(23)	110.85	H(39)-C(27)-C(17)-O(14)	-167.20
H(22)-C(16)	1.09	H(43)-C(25)-C(23)	112.05	H(39)-C(27)-C(17)-O(26)	13.05
C(17)-O(14)	1.37	O(24)-C(23)-C(25)	123.76	H(38)-C(27)-C(17)-O(14)	-45.11
H(15)-C(12)	1.10	O(24)-C(23)-O(20)	118.33	H(38)-C(27)-C(17)-O(26)	135.15
C(16)-C(12)	1.52	C(25)-C(23)-O(20)	117.90	C(23)-O(20)-C(16)-C(12)	-169.76

C(18)-O(11)	1.37	C(23)-O(20)-C(16)	121.67	C(23)-O(20)-C(16)-H(22)	70.24
H(13)-C(10)	1.09	O(30)-C(19)-C(31)	126.06	C(23)-O(20)-C(16)-H(21)	-50.80
O(14)-C(10)	1.44	O(30)-C(19)-O(6)	124.00	C(27)-C(17)-O(14)-C(10)	-170.57
C(12)-C(10)	1.54	C(31)-C(19)-O(6)	109.94	O(26)-C(17)-O(14)-C(10)	9.18
H(9)-C(7)	1.09	O(28)-C(18)-C(29)	123.39	H(22)-C(16)-C(12)-O(4)	-169.30
O(11)-C(7)	1.43	O(28)-C(18)-O(11)	117.86	H(22)-C(16)-C(12)-C(10)	-49.93
C(10)-C(7)	1.53	C(29)-C(18)-O(11)	118.75	H(22)-C(16)-C(12)-H(15)	71.68
C(19)-O(6)	1.37	O(26)-C(17)-C(27)	125.96	H(21)-C(16)-C(12)-O(4)	-49.77
O(6)-C(5)	1.43	O(26)-C(17)-O(14)	123.41	H(21)-C(16)-C(12)-C(10)	69.60
H(8)-C(5)	1.09	C(27)-C(17)-O(14)	110.63	H(21)-C(16)-C(12)-H(15)	-168.79
C(7)-C(5)	1.53	O(20)-C(16)-H(21)	111.64	O(20)-C(16)-C(12)-O(4)	70.86
C(12)-O(4)	1.42	O(20)-C(16)-H(22)	110.30	O(20)-C(16)-C(12)-C(10)	-169.77
H(3)-C(2)	1.11	O(20)-C(16)-C(12)	106.53	O(20)-C(16)-C(12)-H(15)	-48.16
C(5)-C(2)	1.53	H(21)-C(16)-H(22)	108.76	C(29)-C(18)-O(11)-C(7)	6.29
O(4)-C(2)	1.42	H(21)-C(16)-C(12)	109.06	O(28)-C(18)-O(11)-C(7)	-174.27
C(2)-O(1)	1.38	H(22)-C(16)-C(12)	110.54	C(17)-O(14)-C(10)-C(7)	138.38
H(44)-O(1)	0.97	C(17)-O(14)-C(10)	117.53	C(17)-O(14)-C(10)-C(12)	-102.15
		H(15)-C(12)-C(16)	108.47	C(17)-O(14)-C(10)-H(13)	19.76
		H(15)-C(12)-C(10)	109.11	O(4)-C(12)-C(10)-C(7)	-55.53
		H(15)-C(12)-O(4)	110.39	O(4)-C(12)-C(10)-O(14)	-172.34
		C(16)-C(12)-C(10)	113.76	O(4)-C(12)-C(10)-H(13)	66.28
		C(16)-C(12)-O(4)	106.86	C(16)-C(12)-C(10)-C(7)	-174.12
		C(10)-C(12)-O(4)	108.22	C(16)-C(12)-C(10)-O(14)	69.07
		C(18)-O(11)-C(7)	123.65	C(16)-C(12)-C(10)-H(13)	-52.31
		H(13)-C(10)-O(14)	109.22	H(15)-C(12)-C(10)-C(7)	64.62
		H(13)-C(10)-C(12)	110.15	H(15)-C(12)-C(10)-O(14)	-52.19
		H(13)-C(10)-C(7)	110.30	H(15)-C(12)-C(10)-H(13)	-173.57
		O(14)-C(10)-C(12)	111.42	C(18)-O(11)-C(7)-C(5)	-121.57
		O(14)-C(10)-C(7)	105.62	C(18)-O(11)-C(7)-C(10)	116.41
		C(12)-C(10)-C(7)	110.04	C(18)-O(11)-C(7)-H(9)	-2.18
		H(9)-C(7)-O(11)	110.92	C(12)-C(10)-C(7)-C(5)	50.99
		H(9)-C(7)-C(10)	108.22	C(12)-C(10)-C(7)-O(11)	171.27
		H(9)-C(7)-C(5)	108.44	C(12)-C(10)-C(7)-H(9)	-68.43
		O(11)-C(7)-C(10)	108.27	O(14)-C(10)-C(7)-C(5)	171.37
		O(11)-C(7)-C(5)	109.09	O(14)-C(10)-C(7)-O(11)	-68.35
		C(10)-C(7)-C(5)	111.91	O(14)-C(10)-C(7)-H(9)	51.94
		C(19)-O(6)-C(5)	117.31	H(13)-C(10)-C(7)-C(5)	-70.73
		O(6)-C(5)-H(8)	109.63	H(13)-C(10)-C(7)-O(11)	49.55
		O(6)-C(5)-C(7)	107.54	H(13)-C(10)-C(7)-H(9)	169.85
		O(6)-C(5)-C(2)	109.59	C(31)-C(19)-O(6)-C(5)	-177.05
		H(8)-C(5)-C(7)	111.30	O(30)-C(19)-O(6)-C(5)	3.03
		H(8)-C(5)-C(2)	109.02	C(19)-O(6)-C(5)-C(2)	111.50
		C(7)-C(5)-C(2)	109.74	C(19)-O(6)-C(5)-C(7)	-129.26
		C(12)-O(4)-C(2)	113.67	C(19)-O(6)-C(5)-H(8)	-8.12
		H(3)-C(2)-C(5)	110.02	C(10)-C(7)-C(5)-C(2)	-50.91
		H(3)-C(2)-O(4)	108.67	C(10)-C(7)-C(5)-H(8)	69.85
		H(3)-C(2)-O(1)	111.90	C(10)-C(7)-C(5)-O(6)	-170.05
		C(5)-C(2)-O(4)	108.89	O(11)-C(7)-C(5)-C(2)	-170.71
		C(5)-C(2)-O(1)	108.54	O(11)-C(7)-C(5)-H(8)	-49.94

		O(4)-C(2)-O(1)	108.77	O(11)-C(7)-C(5)-O(6)	70.15
		C(2)-O(1)-H(44)	107.79	H(9)-C(7)-C(5)-C(2)	68.38
				H(9)-C(7)-C(5)-H(8)	-170.85
				H(9)-C(7)-C(5)-O(6)	-50.76
				C(10)-C(12)-O(4)-C(2)	65.06
				C(16)-C(12)-O(4)-C(2)	-172.04
				H(15)-C(12)-O(4)-C(2)	-54.28
				C(7)-C(5)-C(2)-O(1)	174.03
				C(7)-C(5)-C(2)-O(4)	55.77
				C(7)-C(5)-C(2)-H(3)	-63.23
				H(8)-C(5)-C(2)-O(1)	51.90
				H(8)-C(5)-C(2)-O(4)	-66.36
				H(8)-C(5)-C(2)-H(3)	174.64
				O(6)-C(5)-C(2)-O(1)	-68.09
				O(6)-C(5)-C(2)-O(4)	173.65
				O(6)-C(5)-C(2)-H(3)	54.65
				C(12)-O(4)-C(2)-O(1)	176.33
				C(12)-O(4)-C(2)-C(5)	-65.55
				C(12)-O(4)-C(2)-H(3)	54.29
				O(4)-C(2)-O(1)-H(44)	-61.69
				C(5)-C(2)-O(1)-H(44)	179.98
				H(3)-C(2)-O(1)-H(44)	58.38
